# Influence and role of polygenic risk score in the development of 32 complex diseases

**DOI:** 10.7189/jogh.15.04071

**Published:** 2025-03-10

**Authors:** Yuxin Liu, Wenyan Hou, Tongyu Gao, Yu Yan, Ting Wang, Chu Zheng, Ping Zeng

**Affiliations:** 1 Department of Biostatistics, School of Public Health, Xuzhou Medical University, Xuzhou, Jiangsu, China; 2 Jiangsu Engineering Research Centre of Biological Data Mining and Healthcare Transformation, Xuzhou Medical University, Xuzhou, Jiangsu, China

## Abstract

**Background:**

The polygenic risk score (PRS) has been perceived as advantageous in predicting the risk of complex diseases compared to other measures. We aimed to systematically evaluate the influence of PRS on disease outcome and to explore its predictive value.

**Methods:**

We comprehensively assessed the relationship between PRS and 32 complex diseases in the UK Biobank. We used Cox models to estimate the effects of PRS on the incidence risk. Then, we constructed prediction models to assess the clinical utility of PRS in risk prediction. For 16 diseases, we further compared the disease risk and prediction capability of PRS across early and late-onset cases.

**Results:**

Higher PRS led to greater incident risk, with hazard ratio (HR) ranging from 1.07 (95% confidence interval (CI) = 1.06–1.08) for panic/anxiety disorder to 4.17 (95% CI = 4.03–4.31) for acute pancreatitis. This effect was more pronounced in early-onset cases for 12 diseases, increasing by 52.8% on average. Particularly, the early-onset risk of heart failure associated with PRS (HR = 3.02; 95% CI = 2.53–3.59) was roughly twice compared to the late-onset risk (HR = 1.48; 95% CI = 1.46–1.51). Compared to average PRS (20–80%), individuals positioned within the top 2.5% of the PRS distribution exhibited varying degrees of elevated risk, corresponding to a more than five times greater risk on average. PRS showed additional value in clinical risk prediction, causing an average improvement of 6.1% in prediction accuracy. Further, PRS demonstrated higher predictive accuracy for early-onset cases of 11 diseases, with heart failure displaying the most significant (37.5%) improvement when incorporating PRS into the prediction model (concordance index (C-index) = 0.546; standard error (SE) = 0.011 *vs.* C-index = 0.751; SE = 0.010, *P* = 2.47 × 10^−12^).

**Conclusions:**

As a valuable complement to traditional clinical risk tools, PRS is closely related to disease risk and can further enhance prediction accuracy, especially for early-onset cases, underscoring its potential role in targeted prevention for high-risk groups.

Over the past two decades, genome-wide association studies (GWASs) have shed light on the important role of numerous single nucleotide polymorphisms (SNPs) in the development of complex diseases [1,2]. Despite the considerably limited impact of each inherited variant on disease risk, they jointly form the genetic basis of many diseases [3]. Based on this, polygenic risk score (PRS), which integrates the genetic information of multiple SNPs, provides a novel perspective for measuring an individual's genetic susceptibility to a specific disease and shows great value for clinical applicability [4,5]. Unlike clinical, biochemistry, and lifestyle risk factors in traditional risk prediction models, PRS rarely alters throughout one's lifetime and can be evaluated in early life, thereby enabling the prompt identification of high-risk individuals [6,7], encouraging lifelong behavioural improvement [8–10], and facilitating targeted interventions [11,12].

Several studies have explored the impact of PRS on various diseases, providing valuable insights into disease susceptibility and progression. For some certain diseases, such as coronary artery disease (CAD) [13], chronic obstructive pulmonary disease (COPD) [14], and atrial fibrillation [15], combining PRS with recognised clinical risk factors has shown superior predictive accuracy. Nevertheless, some investigations have cast doubt on the significance of including PRS [16,17] and its relation with clinical risk factors [18,19], which is why its role continues to be debated [20,21].

Few studies systematically analysed the association between PRS and specific diseases. To fill this gap, we analysed 32 non-communicable diseases (NCDs) available from the UK Biobank. We specifically opted for NCDs, as they are currently responsible for most deaths worldwide and pose a significant public health challenge [22]. Specifically, in addition to major NCDs (*e.g.* cardiovascular diseases, cancer, chronic respiratory diseases, and diabetes), we also incorporated diseases of the digestive, nervous, eye, renal, and immune systems to provide a broader coverage of diverse conditions, while ensuring relatively sufficient cases for each disease. To gain a deeper understanding of the influence and predictive power of PRS, we thoroughly examined the relationship between PRS and disease outcomes and compared the predictive role of PRS with disease-specific clinical risk tools across the new-onset cases and within subgroups of early-onset and late-onset cases for 16 diseases.

## METHODS

### Study population and disease definition

The UK Biobank, a population-based prospective study conducted from 2006 to 2022 [23], was the source of the individual-level data for our analysis. The study collected a comprehensive set of information from over 500 000 people aged 37–73 years nationwide through questionnaires, physical examinations, and biochemical indicators. Individuals of white ethnicity with complete follow-up data for disease diagnosis were included. These diseases can be classified into nine distinct categories, including four neuropsychiatric disorders, two neurodegenerative diseases, seven cardiometabolic diseases, five immune diseases, four digestive diseases, one renal disease, two eye diseases, two respiratory diseases, and five common cancers (Table S1 in the **Online Supplementary Document**).

### Summary data and quality control

To ensure sufficient sample sizes, which in the original GWAS summary statistic ranged from 10 240 for panic/anxiety disorder to 1 308 460 for stroke, we retrieved European ancestry summary statistics of these diseases from the latest GWAS catalogue [24] or FinnGen (R10) [25] (Table S2 in the **Online Supplementary Document**). For each summary statistics data set, we implemented stringent quality control before analyses (**Online Supplementary Document**).

### Calculation of PRS

We calculated the PRS of each disease *via* PRS – continuous shrinkage (PRS-CS) [26], an approach which is advantageous due to its continuous shrinkage before SNP effect sizes, which renders it robust across diverse genetic structures and outperforms other approaches in terms of predictive accuracy (**Online Supplementary Document**). European samples (n = 503) from the 1000 Genomes Project served as the external linkage disequilibrium reference panel [27]. We also calculated the PRS using the pruning and thresholding (P + T) method [28,29] and compared its performance with PRS-CS in predicting disease risk. We discovered that PRS-CS generally showed greater predictive power relative to P + T (Table S3 in the **Online Supplementary Document**). The generated PRS was standardised for association analyses and mostly analysed as a continuous variable.

### Construction of clinical risk tools

Given the impact of clinical factors in forecasting disease risk and comparing the relative contributions of polygenic risk and clinical risk, we incorporated them into models and computed generally recognised clinical risk scores for some diseases (*i.e.* Alzheimer disease [30], hypertension [31], stroke [32], atrial fibrillation [33,34], CAD, myocardial infarction, heart failure [35], and type 2 diabetes (T2D) [36]) to evaluate predictive performance (**Online Supplementary Document**).

For other diseases, we applied available and potential clinical risk factors to predict the risk of occurrence. Considering the specificity of each disease, we reviewed the recent literature and identified possible clinical risk factors, all of which were assessed at baseline to account for their temporal sequence in disease onset and progression. Specifically, we used keywords including ‘clinical risk’, ‘risk prediction’, and ‘Mendelian randomization’ in conjunction with the respective diseases, and then compiled and summarised the common and unique risk factors identified in the retrieved literature (Table S4 in the **Online Supplementary Document**). Missing data in clinical factors were imputed by multivariate imputation by chained equations method [37].

### Statistical analysis

#### Influences of PRS on the risk of incident cases

We identified patients who developed a given disease after recruitment as new-onset cases. The follow-up time of each participant was determined as the period from the baseline to the date of diagnosis, death, or censoring (19 July 2022), whichever occurred first. For new-onset cases of each disease, we used Cox regression, a widely employed in survival analysis, particularly for studying genetic susceptibility and diseases [38–41], to evaluate the impact of continuous or stratified PRS on the incident risk, with the adjustment for disease-specific clinical risk factors and the top ten genetic principal components. Since PRS remains stable throughout life [4], it satisfies the proportional hazards assumption. We reported the hazard ratio (HR) and its 95% confidence intervals (CIs).

To further investigate the independent and combined effects of PRS and clinical risk tools on disease incident risk, we compared cumulative incidence curves based on three models: PRS, clinical risk tools, and PRS combined with clinical risk tools. For the comparison, we categorised incident cases into early-onset and late-onset groups with disease-specific thresholds of early or late onset (Table S5 in the **Online Supplementary Document**).

#### Predictive utility of PRS and clinical risk tools

To evaluate the predictive efficacy of combining clinical risk tools with PRS for new-onset diseases, we established three Cox models with various predictor sets under the Monte Carlo cross-validation framework (80% for training and 20% for validation) [42]: only PRS, clinical risk tools, and PRS + clinical risk tools. We also included the genotype measurement batch and the first ten genetic principal components in clinical risk tools.

We employed the concordance index (C-index) and net reclassification improvement (NRI) to assess the degree of prediction model improvement (**Online Supplementary Document**). Moreover, we used attributable risk to quantify the extent to which the risk was directly attributed to PRS and calculated the number needed to be screened (NNBS) to better illustrate practical application, indicating the number of individuals required to undergo intervention screening to prevent one adverse disease event.

#### Statistical software and packages

We performed all statistical analyses using *R*, version 4.3.1 (R Core Team, Vienna, Austria). *P*-values were two-sided, with the statistical significance set to 1.56 × 10^−3^ (*i.e.* 0.05/32) for multiple testing corrections. We performed the multiple imputations using the R ‘mice’ package, version 3.16.0 [37] and implemented the NRI analysis using the R ‘PredictABEL’ package, version 1.2.4 [43,44].

## RESULTS

### Participants baseline characteristics

**Figure 1:**
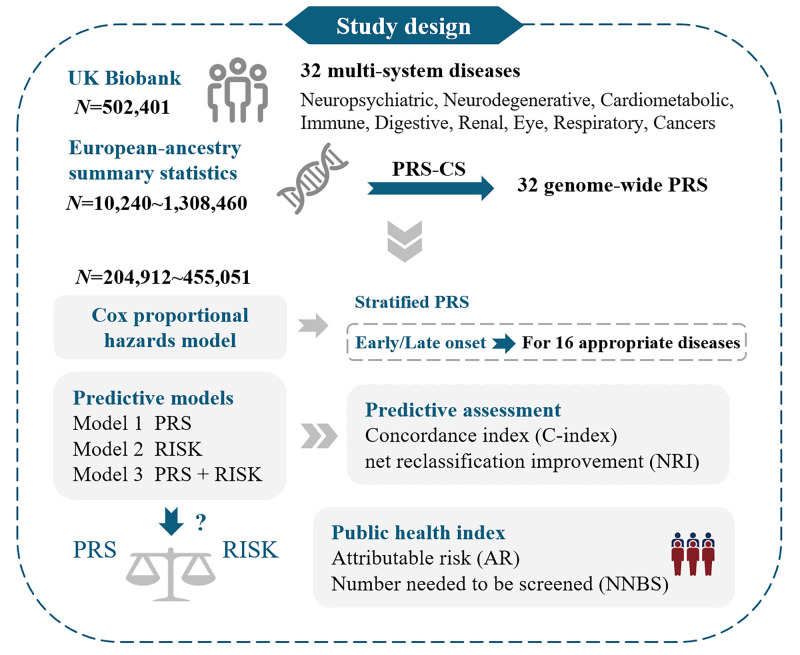
Flowchart of data and analyses for the study. PRS – polygenic risk score, RISK – clinical risk tools.

We included 32 complex diseases, which exhibited varying genetics, prevalences, incidence rates, and median ages at onset (**Figure 1**, **Table 1**). Our analysis included 455 067 white-ancestry participants (Table S6 in the **Online Supplementary Document**). The mean age was 57.3 (standard deviation (SD) = 8.0) and 45.7% were men. Hypertension had the highest incidence rate (18.5%), followed by cataracts (12.6%), gastroesophageal reflux disease (9.8%), and CAD (8.4%). The median age at onset of incident cases varied from 60.6 (interquartile range (IQR) = 53.7–69.1) years for multiple sclerosis to 75.4 (IQR = 71.9–78.1) years for Alzheimer’s disease. We focussed only on women for breast cancer (n = 8799) and men for prostate cancer (n = 10 683).

**Table 1:** Genetic, prevalence, and incidence characteristics of 32 analysed complex diseases*

					Incident	
	**Observed scale, h^2^ (SE)†**	**Liability scale, h^2^ (SE)†**	**Number of independent loci‡**	**Number of SNPs with *P* < 5 × 10^−8^**	**n (%)**	**Age at onset, MD (IQR)**
**Neuropsychiatric disorders**						
PAD	0.292 (0.036)	0.433 (0.053)	0	0	23 205 (5.3)	66.5 (58.6–72.9)
BPD	0.052 (0.002)	0.068 (0.003)	25	808	801 (0.2)	64.6 (56.2–70.8)
MDD	0.030 (0.001)	0.046 (0.002)	48	1785	19 701 (4.7)	65.2 (57.2–72.2)
SCZ	0.272 (0.009)	0.120 (0.004)	163	12 785	428 (0.1)	65.4 (56.2–71.9)
**Neurodegenerative diseases**						
AD	0.017 (0.005)	0.016 (0.005)	30	1953	2989 (0.7)	75.4 (71.9–78.1)
PD	0.013 (0.001)	0.026 (0.002)	21	2700	2662 (0.6)	73.2 (68.9–76.6)
**Cardiometabolic diseases**						
CAD	0.024 (0.001)	0.053 (0.002)	443	12 796	36 105 (8.4)	69.1 (63.4–73.8)
Hypertension	0.094 (0.004)	0.168 (0.007)	276	14 106	61 828 (18.5)	68.3 (61.9–73.2)
Stroke	0.004 (0.000)	0.015 (0.000)	23	739	9503 (2.1)	70.9 (65.5–75.1)
AF	0.018 (0.002)	0.082 (0.009)	113	8889	32 094 (7.2)	71.3 (66.5–75.6)
MI	0.053 (0.004)	0.061 (0.005)	23	767	14 301 (3.2)	69.4 (63.6–74.0)
HF	0.005 (0.001)	0.022 (0.004)	9	217	16 941 (3.7)	72.6 (67.6–76.8)
T2D	0.037 (0.002)	0.069 (0.004)	211	12 137	27 973 (6.3)	68.2 (62.1–72.9)
**Immune diseases**						
Gout	0.021 (0.004)	0.125 (0.024)	11	2056	9255 (2.1)	68.4 (62.2–73.4)
IBD	0.199 (0.019)	0.130 (0.012)	210	7459	3005 (0.7)	66.3 (59.2–71.8)
CD	0.279 (0.030)	0.168 (0.018)	165	6643	1244 (0.3)	65.7 (58.1–71.4)
UC	0.159 (0.015)	0.119 (0.011)	98	2738	2271 (0.5)	66.5 (59.2–71.9)
MS	0.153 (0.032)	0.083 (0.017)	16	429	541 (0.1)	60.6 (53.7–69.1)
**Digestive diseases**						
GERD	0.062 (0.002)	0.113 (0.004)	78	1851	41 209 (9.8)	67.3 (60.6–72.6)
IBS	0.008 (0.001)	0.065 (0.008)	0	0	9362 (2.2)	64.4 (56.7–70.4)
Cholelithiasis	0.040 (0.007)	0.105 (0.018)	64	5207	17 755 (4.0)	66.8 (59.7–72.5)
AP	0.002 (0.000)	0.022 (0.000)	5	25	2833 (0.6)	67.9 (61.0–73.5)
**Renal disease**						
CKD	0.004 (0.001)	0.012 (0.003)	22	944	22 776 (5.1)	71.9 (66.9–76.1)
**Eye diseases**						
Cataract	0.017 (0.001)	0.079 (0.005)	48	1494	55 686 (12.6)	72.3 (67.7–76.1)
Glaucoma	0.134 (0.008)	0.458 (0.027)	101	6208	12 029 (2.7)	70.0 (64.4–74.6)
**Respiratory diseases**						
COPD	0.027 (0.002)	0.110 (0.008)	16	727	15 886 (3.6)	70.2 (65.1–74.3)
Asthma	0.043 (0.006)	0.095 (0.013)	16	588	10 330 (2.6)	67.2 (60.3–72.2)
**Cancer**						
Lung cancer	0.057 (0.009)	0.035 (0.006)	12	1151	4208 (0.9)	69.7 (65.2–73.5)
Breast cancer	0.025 (0.003)	0.145 (0.017)	37	4153	8799 (3.7)	64.8 (57.9–69.7)
Prostate cancer	0.057 (0.008)	0.094 (0.013)	254	26 964	10 683 (5.2)	68.9 (65.0–72.7)
Colon cancer	0.003 (0.001)	0.047 (0.016)	4	125	3894 (0.9)	68.7 (63.3–72.8)
Rectal cancer	0.002 (0.001)	0.047 (0.023)	4	9	1637 (0.4)	67.8 (62.3–72.3)

AD – Alzheimer disease, AF – atrial fibrillation, AP – acute pancreatitis, BPD – bipolar disorder, CAD – coronary artery disease, CD – Crohn's disease, CKD – chronic kidney disease, COPD – chronic obstructive pulmonary disease. GERD – gastroesophageal reflux disease, h^2^ – heritability, HF – heart failure, IBD – inflammatory bowel disease, IBS – irritable bowel syndrome, IQR – interquartile range, MD – median, MDD – major depressive disorder, MI – myocardial infarction, MS – multiple sclerosis, PAD – panic/anxiety disorder, PD – Parkinson disease, SCZ – schizophrenia, SNP – single nucleotide polymorphism, T2D – type 2 diabetes, UC – ulcerative colitis

*The results in the first four columns were derived from summary statistics of each disease, while the remaining results were acquired from the UK Biobank.

†h^2^ on the observed scale was computed through linkage disequilibrium score regression and the h^2^ on the liability scale was calculated *via* the method proposed by Lee and colleagues [45].

‡The identification of independent loci was facilitated by functional mapping and annotation of genome-wide association studies.

### Estimated impacts of PRS and incident risk of complex diseases

#### Incidence of complex diseases by percentile PRS

We observed a trend of increasing incidence as PRS elevated for these diseases with varying magnitudes (Figure S1 in the **Online Supplementary Document**). The average disparity in the incidence rates between the highest (>99%) and lowest (<1%) PRSs was 9.7%, with the most pronounced variation observed for cataracts (29.1%) and the smallest increase for multiple sclerosis (0.2%).

#### Association between continuous PRS and incident risk of complex diseases

For all these diseases, PRS had a notable effect on the risk of incidence, with HR ranging from 1.07 (95% CI = 1.06–1.08) for panic/anxiety disorder to 4.17 (95% CI = 4.03–4.31) for acute pancreatitis, and the average elevated risk was 66% across these diseases. Apart from acute pancreatitis, PRS also showed greater impacts on the occurrence risk of other diseases (**Table 2**; Table S7 in the **Online Supplementary Document**) – for instance, colon cancer (HR = 3.32; 95% CI = 3.24–3.41) and rectal cancer (HR = 3.09; 95% CI = 3.01–3.18).

**Table 2:** Incident risk association for PRS (per SD) for 32 complex diseases

	All incident	
	**HR (95% CI)**	***P-*value**
**Neuropsychiatric disorders**		
PAD	1.07 (1.06–1.08)	9.61 × 10^−25^
BPD	1.46 (1.36–1.56)	4.18 × 10^−26^
MDD	1.27 (1.25–1.28)	1.44 × 10^−238^
SCZ	1.66 (1.50–1.83)	6.60 × 10^−24^
**Neurodegenerative diseases**		
AD	1.96 (1.90–2.02)	<0.001
PD	1.33 (1.28–1.38)	1.21 × 10^−47^
**Cardiometabolic diseases**		
CAD	1.46 (1.44–1.47)	<0.001
Hypertension	1.27 (1.26–1.28)	<0.001
Stroke	1.49 (1.46–1.52)	<0.001
AF	1.66 (1.64–1.68)	<0.001
MI	1.34 (1.32–1.36)	8.51 × 10^−264^
HF	1.49 (1.47–1.51)	<0.001
T2D	1.87 (1.85–1.89)	<0.001
**Immune diseases**		
Gout	1.37 (1.34–1.39)	4.65 × 10^−195^
IBD	1.41 (1.36–1.46)	5.94 × 10^−77^
CD	1.48 (1.40–1.57)	1.46 × 10^−43^
UC	1.42 (1.36–1.48)	8.00 × 10^−61^
MS	1.36 (1.25–1.48)	6.42 × 10^−13^
**Digestive diseases**		
GERD	1.37 (1.36–1.38)	<0.001
IBS	1.12 (1.10–1.14)	1.10 × 10^−27^
Cholelithiasis	1.38 (1.36–1.40)	<0.001
AP	4.17 (4.03–4.31)	<0.001
**Renal disease**		
CKD	1.23 (1.21–1.24)	1.09 × 10^−203^
**Eye diseases**		
Cataract	1.49 (1.47–1.50)	<0.001
Glaucoma	1.78 (1.75–1.81)	<0.001
**Respiratory diseases**		
COPD	1.26 (1.24–1.28)	8.28 × 10^−186^
Asthma	1.13 (1.11–1.16)	1.50 × 10^−36^
**Cancer**		
Cancer	1.29 (1.25–1.33)	1.91 × 10^−61^
Lung cancer	2.16 (2.12–2.21)	<0.001
Breast cancer	2.09 (2.05–2.13)	<0.001
Prostate cancer	3.32 (3.24–3.41)	<0.001
Colon cancer	3.09 (3.01–3.18)	<0.001

AD – Alzheimer disease, AF – atrial fibrillation, AP – acute pancreatitis, BPD – bipolar disorder, CAD – coronary artery disease, CD – Crohn's disease, CI – confidence interval, CKD – chronic kidney disease, COPD – chronic obstructive pulmonary disease, GERD – gastroesophageal reflux disease, HF – heart failure, HR – hazard ratio, IBD – inflammatory bowel disease, IBS – irritable bowel syndrome, MDD – major depressive disorder, MI – myocardial infarction, MS – multiple sclerosis, PAD – panic/anxiety disorder, PD – Parkinson disease, PRS – polygenic risk score, SCZ – schizophrenia, SD – standard deviation, T2D – type 2 diabetes, UC – ulcerative colitis

For certain diseases such as CAD, hypertension, myocardial infarction, T2D, and gout, PRS showed a higher cumulative incidence compared to clinical risk tools alone. The combined effect of PRS and clinical risk tools was particularly pronounced in mid to late life for inflammatory bowel disease, Crohn's disease, ulcerative colitis, multiple sclerosis, and asthma (Figure S2 in the **Online Supplementary Document**).

#### Association between continuous PRS and early or late onset of complex diseases

**Figure 2:**
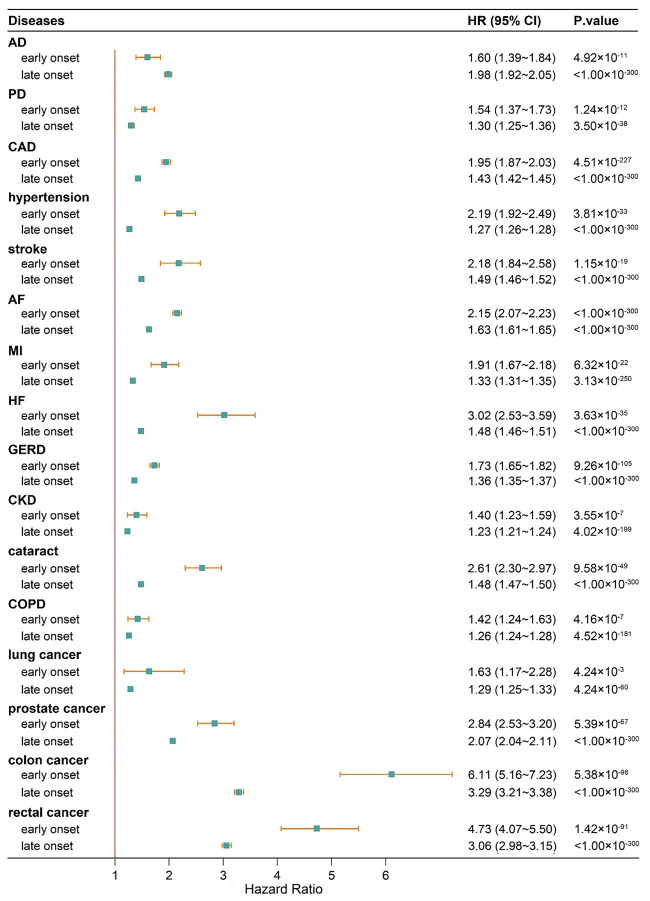
Influence of PRS on disease risk on the early-onset or late-onset cases for 16 diseases. AD – Alzheimer disease, AF – atrial fibrillation, CAD – coronary artery disease, CI – confidence interval, CKD – chronic kidney disease, COPD – chronic obstructive pulmonary disease, GERD – gastroesophageal reflux disease, HF – heart failure, HR – hazard ratio, MI – myocardial infarction, PD – Parkinson disease.

Given the age of enrollment in the UK Biobank and the cut-off age for early disease onset, we ultimately examined the influence of PRS on 16 diseases (**Figure 2**). For all diseases except for Alzheimer disease, chronic kidney disease, COPD, and lung cancer, the early-onset risk attributed to PRS was higher than the late-onset risk, with the effect size increased by 52.8% on average. For instance, the early-onset risk of heart failure associated with PRS (HR = 3.02; 95% CI = 2.53–3.59) was roughly twice compared to the late-onset risk (HR = 1.48; 95% CI = 1.46–1.51), while the difference between the early-onset risk (HR = 1.42; 95% CI = 1.24–1.63) and the late-onset risk (HR = 1.26; 95%CI = 1.24–1.28) due to PRS on COPD was rather marginal, amounting to a mere 12% increase (Table S7 in the **Online Supplementary Document**).

#### Association between stratified PRS and incident risk of complex diseases

We categorised the PRS into multiple levels (<2.5%, 2.5–20%, 20–80%, 80–97.5%, and >97.5%), with 20–80% as the reference to compare the risk of individuals to those with an average risk. The choice of the top and bottom 2.5% follows a similar rationale to laboratory test reference ranges, where values that encompass 95% of the population are typically used as the standard range. After adjusting for clinical risk tools, individuals within the top 2.5% of the PRS distribution showed varying degrees of elevated risk in comparison with those with average PRS (20–80%), with the HR ranging from 1.26 (95% CI = 1.17–1.36) for panic/anxiety disorder to 36.27 (95% CI = 32.94–39.93) for acute pancreatitis (Table S8 in the **Online Supplementary Document**), corresponding to a more than five times higher risk on average. Similarly, compared with average PRS, those in the bottom 2.5% of the PRS distribution had a lower risk, with HR varying from 0.11 (95% CI = 0.07–0.16) for prostate cancer to 0.77 (95% CI = 0.72–0.82) for hypertension, corresponding to an average HR of 0.56, indicating a reduced risk across various diseases.

### Performance of PRS for the prediction of complex diseases

#### Predictive efficiency of distinct models

When PRS was the sole factor considered in the prediction model, the predictive performance of acute pancreatitis peaked at an impressive C-index of 0.835 (standard error (SE) = 0.002), while panic/anxiety disorder had the lowest C-index of 0.517 (SE *=* 0.001). Integrating PRS into the model that only included clinical risk tools significantly enhanced prediction accuracy (Table S9 in the **Online Supplementary Document**). Typically, the model incorporating both PRS and clinical risk tools had an average improvement of 6.1% in prediction accuracy. Among all diseases, the predictive ability of PRS for acute pancreatitis showed the greatest improvement of 31.1% (C-index = 0.846, SE = 0.002 *vs.* C-index = 0.645, SE = 0.004; *P* = 7.35 × 10^−19^). Particularly, the predictive performance was improved by 26.2% (*P* = 2.67 × 10^−23^) for breast cancer, 13.9% (*P* = 6.26 × 10^−12^) for rectal cancer, 13.5% (*P* = 7.42 × 10^−10^) for Crohn’s disease, 13.1% (*P* = 1.74 × 10^−14^) for colon cancer, and 11.0% (*P* = 3.85 × 10^−22^) for prostate cancer, indicating a notable contribution of PRS in refining risk prediction of these diseases. The improvement in predictive efficacy for gout remained minimal at a 1.5% increase (*P* = 0.23).

#### Efficacy of PRS in predicting early-onset and late-onset diseases

Compared to the model that only included clinical risk tools, the model additionally incorporating PRS demonstrated a greater predictive ability for each analysed disease in both early-onset and late-onset individuals, with heart failure having the most significant (37.5%) improvement among early-onset cases (C-index = 0.546, SE = 0.011 *vs.* C-index = 0.751, SE = 0.010; *P* = 2.47 × 10^−12^), and colon cancer the most notable (12.7%) improvement in late-onset cases (C-index = 0.687, SE = 0.002 *vs.* C-index = 0.774, SE = 0.003; *P* = 6.29 × 10^−16^) (Table S9 in the **Online Supplementary Document**).

**Figure 3:**
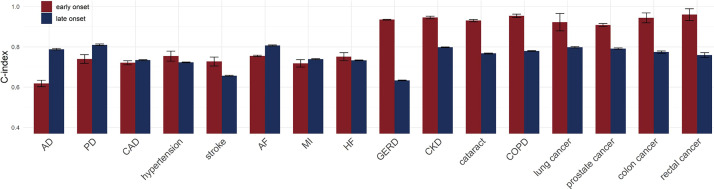
Comparison of C-index for the model that simultaneously included PRS and clinical risk tools in predicting early-onset and late-onset disease cases. AD – Alzheimer disease, AF – atrial fibrillation, CAD – coronary artery disease, CKD – chronic kidney disease, COPD – chronic obstructive pulmonary disease, GERD – gastroesophageal reflux disease, HF – heart failure, MI – myocardial infarction, PD – Parkinson disease.

Comparing the predictive ability of the model that simultaneously included PRS and clinical risk tools in early-onset and late-onset cases, the performance varied across different diseases (**Figure 3**). For Alzheimer disease, Parkinson disease, CAD, atrial fibrillation, and heart failure, the model exhibited superior predictive efficacy in the late-onset cases compared to the early-onset group, with Alzheimer disease showing the most significant (21.5%) improvement (C-index = 0.618, SE = 0.008 *vs.* C-index = 0.788, SE = 0.002; *P* = 9.79 × 10^−11^). Particularly, for the remaining 11 complex diseases, the model showed a stronger predictive ability in the early-onset cases (Table S9 in the **Online Supplementary Document**), Gastroesophageal reflux disease had the greatest improvement, achieving a 47.8% increase (C-index = 0.935, SE = 0.001 *vs.* C-index = 0.633, SE = 0.001; *P* = 8.40 × 10^−40^).

#### Quantification of risk reduction and prevention for 32 diseases

For all the 32 diseases, the model with additional PRS demonstrated a superior ability to predict disease occurrence compared to the model without PRS (all NRI>0) (Table S10 in the **Online Supplementary Document**). For most analysed diseases, the NRI for early-onset cases was significantly higher than that for late-onset individuals. We also detected a relatively large change for hypertension (14.6 times higher) and stroke (13.0 times higher).

Lastly, we calculated the absolute risk reduction and NNBSs to understand the significance of PRS for disease prevention (**Table 3**). Multiple sclerosis required the largest NNBS (n = 763) to prevent one adverse outcome for populations having the PRS in the top 2.5% of the PRS distribution. Conversely, cataracts had the lowest NNBS, with only approximately five people to be screened within populations in the top 2.5% of the PRS distribution to achieve the same preventive effect.

**Table 3:** NNBS for 32 complex diseases*

	ARR, %	NNBS, n†
**Neuropsychiatric disorders**		
PAD	1.3	75
BPD	0.3	357
MDD	3.2	31
SCZ	0.2	520
**Neurodegenerative diseases**		
AD	2.8	36
PD	0.6	164
**Cardiometabolic diseases**		
CAD	10.8	9
Hypertension	11.5	9
Stroke	4.2	24
AF	14.3	7
MI	2.9	34
HF	6.8	15
T2D	15.8	6
**Immune diseases**		
Gout	1.6	61
IBD	0.7	144
CD	0.4	246
UC	0.7	152
MS	0.1	763
**Digestive diseases**		
GERD	8.9	11
IBS	0.6	172
Cholelithiasis	4.5	22
AP	8.8	11
**Renal disease**		
CKD	3.0	33
**Eye diseases**		
Cataract	18.3	5
Glaucoma	7.1	14
**Respiratory diseases**		
COPD	2.5	40
Asthma	0.9	114
**Cancer**		
Lung cancer	0.9	112
Breast cancer	15.4	6
Prostate cancer	16.4	6
Colon cancer	11.9	8
Rectal cancer	5.6	18

ARR – absolute risk reduction, AD – Alzheimer disease, AF – atrial fibrillation, AP – acute pancreatitis, BPD – bipolar disorder, CAD – coronary artery disease, CD – Crohn's disease, CKD – chronic kidney disease, COPD – chronic obstructive pulmonary disease, GERD – gastroesophageal reflux disease, HF – heart failure, IBD – inflammatory bowel disease, IBS – irritable bowel syndrome, MDD – major depressive disorder, MI – myocardial infarction, MS – multiple sclerosis, NNBS – number needed to be screened, PAD – panic/anxiety disorder, PD – Parkinson disease, PRS – polygenic risk score, SCZ – schizophrenia, T2D – type 2 diabetes, UC – ulcerative colitis

*Stratified PRS for high PRS (the top 2.5% of the distribution) and low PRS (the rest of the distribution).

†NNBS = 1/ARR.

## DISCUSSION

We assessed the relationship between PRS and 32 complex diseases and discovered two key insights. First, higher PRS caused higher incident risk, which was more pronounced in early-onset cases compared to late-onset cases, with the effect size increasing by more than 50% on average. Second, PRS demonstrated added value in clinical risk prediction, especially for early-onset cases, and its inclusion improved the reclassification of individuals.

### Comparison to previous studies

#### Association analyses

Previous studies examined the association between PRS and a few diseases [46–49], yet most used a small subset of significant SNPs to develop PRS, relied on cross-sectional data, or involved relatively limited sample sizes. Conversely, we included 32 diseases across nine categories and assessed the complex relationship between genome-wide PRS and diseases in a prospective cohort with a sufficiently large sample size and a longer follow-up period. In the new-onset cases, we allowed for disease-specific clinical risk factors, evaluated the impact of PRS on both early-onset and late-onset cases, and further compared the predictive performance of PRS with clinical risk tools, offering deeper insights into the precise use of PRS at different life stages.

Our results align with previous studies [50–52], showing that a higher PRS is associated with an increased disease risk. This correlation remained significant after considering various potential clinical confounders. We extended and reinforced the findings of a recent study which considered only five diseases and 135 300 participants [50], while we included 32 diseases in a larger cohort (n = 455 067).

Given the enrollment age of the UK Biobank and the cut-off age for early disease onset, we specifically focussed on 16 diseases. We found that most exhibited a higher early-onset risk, aligning with some prior findings [53–55]. However, for Alzheimer disease, chronic kidney disease, COPD, and lung cancer, the late-onset risk was significantly greater. There are some explanations for this observation. Specifically, as a neurodegenerative disease, Alzheimer disease is typically associated with age, and its late-onset variant is far more common than its early-onset one [56]. Similarly, chronic kidney disease, COPD, and lung cancer tend to manifest later in life due to the cumulative effects of long-term exposure to environmental factors, such as air pollution and smoking, or the gradual decline in organ function over time [57,58]. Consequently, the effect of PRS may be masked in these conditions.

#### Predictive performance

The predictive value of PRS and clinical risk tools varied by specific diseases. Although PRS alone cannot definitively predict the diagnosis of complex diseases as genetic factors contributed only a portion of the risk and PRS captured only part of genetic contributions [59], the integration of PRS into clinical risk prediction models significantly improved predictive accuracy and refined risk reclassification, consistent with previous findings [60–62].

However, existing studies have paid limited attention to the impact of PRS on predicting the age at onset of diseases [52,63,64]. Here we further focussed on both early-onset and late-onset cases. When examined alone, PRS generally showed improved predictive performance for early-onset individuals, except in the case of Alzheimer disease. This suggests that PRS has the potential to offer valuable insights into disease risk early in life. Moreover, incorporating PRS into clinical risk tools generally improved reclassification performance for early-onset cases, providing further evidence for a wider range of diseases compared to prior studies [50,65].

### Clinical and public health implications

Given the substantial socioeconomic and health burden of NCDs worldwide, early identification of high-risk individuals is crucial for reducing disease progression and mortality [66]. We systematically analysed the association and predictive performance of PRS for 32 complex diseases, thereby addressing a significant portion of the NCD burden.

Genetic factors are crucial in disease susceptibility, and PRS provides the means to quantify this risk and shows promise in predicting disease occurrence early in life, even before clinical risk factors emerge [67]. For example, it can be used in early screening for CAD in high-risk individuals, enabling health care providers to identify patients who may benefit from earlier interventions, such as lifestyle changes or preventive treatments, long before traditional risk factors manifest (*e.g.* hypertension and cholesterol). In addition, the results of NNBS can offer specific information to balance risk, cost, and benefit in targeting effective screening for those at high risk [68]. For example, in the case of CAD, preventing one adverse outcome in a population would require screening approximately nine individuals in the top 2.5% of the PRS distribution. Advancements in sequencing technology have made genetic information more accessible and versatile, allowing for its application across a wide range of purposes [69]. Despite the practical challenges in integrating genetic information into clinical decision-making, the combined use of PRS and clinical risk tools provided a more comprehensive risk assessment by incorporating genetic and non-genetic factors.

Diseases that manifest at an early age are often underdiagnosed, leading to poor clinical outcomes and even irreversible damage [70–72]. This highlights the urgency of incorporating PRS into early screening programmes to identify high-risk individuals before disease onset and to promote lifestyle modifications. An integrated approach is crucial for developing personalised prevention and treatment strategies, optimising patient prognosis, and advancing precision medicine [73]. Our findings further highlight the need for targeted prevention and intervention strategies tailored to the specific risk profiles of each disease, accounting for diverse aetiologies and disease progression patterns at different life stages [4].

While PRS holds great promise for personalised medicine and early intervention, its implementation must be carefully managed to prevent genetic discrimination, where individuals may face unfair treatment or restricted access to certain services based on their genetic risk. Therefore, establishing ethical guidelines and policies to regulate PRS use is essential. Additionally, ensuring transparency in how genetic data are used and communicating the limitations and scope of PRS will be crucial for maintaining public trust in its application.

### Strength, limitations, and future research

Our study has several strengths. First, we leveraged recent and large-scale summary statistics and applied PRS-CS, which has proven to be more robust than other methods by capturing a broader spectrum of genetic information [26]. Second, we significantly extended previous research by comprehensively evaluating the association between PRS and 32 complex diseases within the same population. This approach ensured greater comparability in terms of study design, disease definition, and other factors, allowing us to assess the generalisability of PRS across diverse health outcomes and identify common risk patterns among diseases. Furthermore, by comparing PRS with disease-specific risk factors, we demonstrated its incremental predictive value beyond traditional risk factors, highlighting its potential to refine disease risk stratification and support more personalised approaches to disease prevention and management.

However, our study also has several limitations. First, the UK Biobank primarily consists of individuals of European ancestry and includes volunteers who tend to be healthier than the general population [74]. Additionally, the average recruitment age is around 40 years, limiting our ability to analyse diseases with an earlier onset (*e.g.* T2D). Second, while PRS exhibited higher utility in individuals of European ancestry [75], caution is needed when extrapolating these findings to other ethnic groups [76,77]. However, focussing on a single ancestry group ensures a more homogeneous genetic background, leading to more accurate and interpretable results. A crucial future direction is to explore the association between multi-ancestry PRS and disease risk [78]. Additionally, baseline clinical risk factors may change over time, potentially confounding the effects of PRS. Future studies should incorporate more nuanced time-series analyses when comparing PRS with clinical risk tools and work towards developing novel clinical risk assessment models.

## CONCLUSIONS

Our results showed that individuals with higher PRS face greater disease risk, highlighting its role for targeted prevention in high-risk groups. The approach could thus complement traditional clinical risk tools, significantly enhancing the overall accuracy and reliability of clinical risk prediction, especially for early-onset cases.

## Additional material


Online Supplementary Document
