# Supplementary material for: Influence and role of polygenic risk score in the development of 32 complex diseases
Source: J Glob Health. 2025 Mar 10;15:04071. doi: 10.7189/jogh.15.04071 (PMC11893022; doi:10.7189/jogh.15.04071)
Supplement: Online Supplementary Document [file jogh-15-04071-s001.pdf]

Supplement to: Liu Y, Hou W, Gao T, Yan Y, Wang T, Zheng C, Zeng P. Influence and role of polygenic risk score in the development of 32 complex diseases. J Glob Health. 2025;15:04071.

Disease definitions

Most of diseases endpoints were identified by first occurrence records (Category 1712), which were obtained through the Primary Care data (Category 3000), Hospital inpatient data (Category 2000), Death Register records (Fields 40001 and 40002) and self-reported medical condition codes (Field 20002). Specifically, for Alzheimer's disease (AD), Parkinson's disease (PD), stroke, myocardial infarction (MI), chronic obstructive pulmonary disease (COPD) and asthma, algorithmically-defined outcomes (Category 42) were used to ascertain events, combining with coded information gathered from self-reported conditions, hospital admissions and death registries. To confirm the incidence of cancers, we utilized the applicable ICD9 and ICD10 definition from national cancer registries (Fields 40006, 40008 and 40013). Detailed fields and codes of these diseases in the UK Biobank cohort are shown in Table S1.

Quality control of summary statistics

For each summary statistics dataset, we implemented stringent quality control procedures for disease: (i) deleted SNPs with missing and duplicated rs IDs; (ii) removed all non-biallelic SNPs; (iii) excluded SNPs with strand-ambiguous alleles (SNPs with A/T, C/G alleles); (iv) eliminated SNPs not present in the reference panel [1]; (v) excluded SNPs located within the major histocompatibility complex (MHC) region (chr6: 28.5-33.5 Mb) due to its complicated linkage disequilibrium (LD) structure [2]; (vi) retained SNPs with a minor allele frequency (MAF) > 0.01. For unavailable MAF information in some summary statistics, we estimated it by applying the genotype data of European sample in the 1000 Genomes Project.

Polygenic risk score

For each disease, we calculated its polygenic risk score (PRS) via polygenic risk scores-continuous shrinkage (PRS-CS) [3]. This approach offers several advantages, including robustness across diverse genetic architectures, significant computational efficiency, and the ability to model local LD patterns. Unlike other methods, such as pruning and thresholding (P+T), which rely on a small set of pre-selected, uncorrelated significant

SNPs, PRS-CS utilizes genome-wide variants. This minimizes subjectivity in locus selection and threshold determination, facilitating flexible adjustments, especially in situations involving fewer and weaker associated SNPs.

Moreover, compared to LDpred which employs a point-normal mixture prior on genetic effect sizes [4], PRS-CS leverages a Bayesian regression framework that utilizes a continuous shrinkage (CS) prior on SNP effect sizes [3]. The CS prior enables adaptive shrinkage, adjusting the level of shrinkage applied to each SNP based on its association strength in genome-wide association studies (GWASs). Additionally, the CS prior supports conjugate block updates during posterior inference, where the SNP effect sizes within each LD block are updated jointly in a multivariate manner, rather than sequentially and individually for each marker. This approach allows for accurate modelling of local LD patterns and significantly enhances computational efficiency. Further, PRS-CS requires only GWAS summary statistics and an external LD reference panel, making it applicable in a wider array of contexts.

We also calculated the PRS for each disease using the P+T method and compared its performance with PRS-CS in predicting disease risk. Specifically, logistic regression was utilized to assess the relationship between PRS and disease risk, adjusting for gender (except for breast and prostate cancer) and the top ten principal components. As shown in Table S3, PRS-CS generally outperformed P+T in terms of predictive accuracy, with higher R^2. Notably, for diseases like colon and rectal cancer, PRS-CS showed particularly strong predictive power. Based on these results, PRS-CS was selected to calculate the PRS for all the diseases analyzed.

Clinical risk score

Specifically, for Alzheimer's disease (AD), we employed the Cardiovascular Risk Factors, Ageing and Dementia (CAIDE) risk score [5], a comprehensive approach that included age, sex, education, systolic blood pressure (SBP), body mass index (BMI), total cholesterol (TC), physical activity and apolipoprotein ε4 (APOE4), each variable assigned respective weights. The CAIDE score has been validated to assess the risk of dementia two decades later, with higher score indicating an increased likelihood of future dementia onset.

For hypertension, the Framingham Hypertension Risk Score (FRS-HPT) was utilized

to evaluate the short-term risk of developing hypertension [6]. FRS-HPT was a well-known predictive model, comprising age, sex, systolic blood pressure (SBP) and diastolic blood pressure (DBP), body mass index (BMI), parental hypertension, and cigarette smoking, which has been demonstrated good efficacy in estimating the four-year risk of hypertension onset.

For stroke, we considered clinical risk using a novel stroke risk stratification technique, the CHA_2DS_2-VASc score [7], which totaled points for the following: one point for congestive heart failure, hypertension, age 65-74 years, diabetes, female and vascular diseases, and two points for age ≥75 years and history of stroke, transient ischemic attack (TIA) or thromboembolism (TE). Vascular disease was defined as history of myocardial infarction, complex aortic plaque, or peripheral artery disease.

Clinical risk score for atrial fibrillation (AF) was calculated using the CHARGE-AF [8,9], a 5-year risk prediction model generated from three large cohorts, incorporating age, height, weight, systolic blood pressure (SBP) and diastolic blood pressure (DBP), cigarette smoking, previous use of antihypertensive medications, diabetes, and history of myocardial infarction (MI) and heart failure (HF).

For CAD, MI and HF, the Framingham Risk Score (FRS) served as a valid algorithm for predicting the 10-year risk of cardiovascular disease development [10], which considered various factors, consisting of age, sex, total and high-density lipoprotein cholesterol, systolic blood pressure, treatment for hypertension, cigarette smoking and diabetes status.

For type 2 diabetes (T2D), we performed a 10-year risk score that based on the latest ADA criteria [11], which included accessible variables: age, sex, history of gestational diabetes for females, family history of any diabetes, hypertension, physical activity, weight and height (Table S3).

Net reclassification improvement

Net reclassification improvement (NRI) [12] is a statistical method used to quantify the ability of a new model to correctly classify individuals into risk categories. In our study, it was selected to evaluate the improvement in predictive accuracy when combining PRS with clinical risk tools, offering additional insights about the clinical utility of PRS

in risk stratification. The NRI measures the net change in the correct classification of individuals, capturing both individuals who are reclassified into higher or lower risk categories and those who are misclassified. An NRI greater than zero indicates that the new model has a better classification performance than the original model.

Table S1. Complex diseases endpoint definitions.

Category	Diseases	Definitions
Neuropsychiatric disorders	PAD	Fields for first occurrence: 130904, 130906
	BPD	Fields for first occurrence: 130894
	MDD	Fields for first occurrence: 130896, 130898
	SCZ	Fields for first occurrence: 130874, 130876
Neurodegenerative diseases	AD	Field 42020
	PD	Field 42032
Cardiometabolic diseases	CAD	Fields for first occurrence: 131296, 131298, 131300, 131302, 131304, 131306
	hypertension	Fields for first occurrence: 131286, 131288, 131290, 131292, 131294
	stroke	Field 42006
	AF	Fields for first occurrence: 131350
	MI	Field 42000
	HF	Fields for first occurrence: 131354
	T2D	Fields for first occurrence: 130708
Immune diseases	gout	Fields for first occurrence: 131858
	IBD	Fields for first occurrence: 131626, 131628
	CD	Fields for first occurrence: 131626
	UC	Fields for first occurrence: 131628
	MS	Fields for first occurrence: 131042
Digestive diseases	GERD	Fields for first occurrence: 131584
	IBS	Fields for first occurrence: 131638
	cholelithiasis	Fields for first occurrence: 131674
	AP	Fields for first occurrence: 131682
Renal disease	CKD	Fields for first occurrence: 132032
Eye diseases	cataract	Fields for first occurrence: 131164, 131166
	glaucoma	Fields for first occurrence: 131186, 131188
Respiratory diseases	COPD	Field 42016
	asthma	Field 42014

Cancer	lung cancer	ICD9: 1623, 1624, 1625, 1628, 1629; ICD10: C34
	breast cancer	ICD9: 174; ICD10: C50
	prostate cancer	ICD9: 1859; ICD10: C61
	colon cancer	ICD9: 153; ICD10: C18
	rectal cancer	ICD9: 154; ICD10: C20

Note: PAD, panic/anxiety disorder; BPD, bipolar disorder; MDD, major depressive disorder; SCZ, schizophrenia; AD, Alzheimer's disease; PD, Parkinson disease; CAD, coronary artery disease; AF, atrial fibrillation; MI, myocardial infarction; HF, heart failure; T2D, type 2 diabetes; IBD, inflammatory bowel disease; CD, Crohn's disease; UC, ulcerative colitis; MS, multiple sclerosis; GERD, gastroesophageal reflux disease; IBS, irritable bowel syndrome; AP, acute pancreatitis; CKD, chronic kidney disease; COPD, chronic obstructive pulmonary disease.

Table S2. Summary data used for construction of polygenic risk score (PRS).

Category	Diseases	Original SNPs	SNPs in PRS calculation	*N* (cases/controls)	Reference
Neuropsychiatric disorders	PAD	10,151,624	6,248,119	10,240 (2,248/7,992)	[13]
	BPD	4,623,182	3,546,755	413,466 (41,917/371,549)	[14]
	MDD	7,266,506	5,655,774	674,452 (166,773/507,679)	[15]
	SCZ	7,659,767	5,843,918	130,644 (53,386/77,258)	[16]
Neurodegenerative diseases	AD	10,687,077	6,268,901	472,868 (75,024/397,844)	[17]
	PD	17,510,617	6,095,495	482,730 (33,674/449,056)	[18]
Cardiometabolic diseases	CAD	12,763,516	6,723,268	1,165,690 (181,522/984,168)	[19]
	hypertension	5,265,189	4,179,283	458,554 (144,793/313,761)	[20]
	stroke	7,511,476	5,786,521	1,308,460 (73,652/1,234,808)	[21]
	AF	34,740,186	6,562,834	1,030,836 (60,620/970,216)	[22]
	MI	8,469,492	6,063,215	166,065 (42,561/123,504)	[23]
	HF	8,274,408	5,979,553	977,323 (47,309/930,014)	[24]
	T2D	18,409,439	6,101,246	298,957 (48,286/250,671)	[25]
Immune diseases	gout	21,301,862	6,323,026	272,412 (9,568/262,844)	[26]
	IBD	23,101,902	5,817,129	59,957 (25,042/34,915)	[27]
	CD	23,077,455	5,788,193	40,266 (12,194/28,072)	[27]
	UC	23,056,709	5,792,645	45,975 (12,366/33,609)	[27]
	MS	7,968,107	5,464,784	15,283 (4,888/10,395)	[28]
Digestive diseases	GERD	2,324,711	1,803,334	602,604 (129,080/473,524)	[29]
	IBS	21,304,585	6,323,026	339,710 (10,329/329,381)	[26]
	cholelithiasis	21,306,148	6,323,026	401,832 (40,191/361,641)	[26]
	AP	9,842,983	5,910,726	855,309 (10,630/844,679)	[30]
Renal disease	CKD	9,162,323	6,135,996	480,698 (41,395/439,303)	[31]
Eye diseases	cataract	9,869,154	5,990,423	459,936 (31,852/428,084)	[32]
	glaucoma	8,002,429	6,082,469	127,265 (7,947/119,318)	[33]
Respiratory diseases	COPD	21,305,215	6,323,026	358,369 (20,066/338,303)	[26]

	asthma	2,001,280	1,805,398	127,669 (19,954/107,715)	[34]
Cancer	lung cancer	7,884,164	5,750,553	85,716 (29,266/56,450)	[35]
	breast cancer	9,969,821	6,323,486	428,231 (17,881/410,350)	[36]
	prostate cancer	27,221,027	6,685,618	726,828 (122,188/604,640)	[37]
	colon cancer	9,987,214	6,330,679	414,143 (3,793/410,350)	[36]
	rectal cancer	9,987,289	6,330,721	412,441 (2,091/410,350)	[36]

Note: PAD, panic/anxiety disorder; BPD, bipolar disorder; MDD, major depressive disorder; SCZ, schizophrenia; AD, Alzheimer's disease; PD, Parkinson disease; CAD, coronary artery disease; AF, Atrial fibrillation; MI, myocardial infarction; HF, heart failure; T2D, type 2 diabetes; IBD, inflammatory bowel disease; CD, Crohn's disease; UC, ulcerative colitis; MS, multiple sclerosis; GERD, gastroesophageal reflux disease; IBS, irritable bowel syndrome; AP, acute pancreatitis; CKD, chronic kidney disease; COPD, chronic obstructive pulmonary disease. Summary statistics of gout, IBS, cholelithiasis and COPD derived from FinnGen (R10).

Table S3. P+T vs. PRS-CS in predicting disease risk.

Category	Diseases	Total SNPs	R^2_{P+T}	$R^2_{PRS\text{-}CS}$
Neuropsychiatric disorders	PAD*	26	0.0101	0.0105
	BPD	25	0.0021	0.0148
	MDD	50	0.0081	0.0167
	SCZ	187	0.0162	0.0403
Neurodegenerative diseases	AD	54	0.0163	0.0492
	PD	26	0.0126	0.0190
Cardiometabolic diseases	CAD	535	0.0358	0.0686
	hypertension	359	0.0205	0.0771
	stroke	23	0.0089	0.0189
	AF	146	0.0314	0.0685
	MI	26	0.0479	0.0591
	HF	10	0.0181	0.0437
	T2D	261	0.0277	0.0912
Immune diseases	gout	28	0.0798	0.0933
	IBD	252	0.0083	0.0282
	CD	203	0.0067	0.0296
	UC	114	0.0088	0.0276
	MS	17	0.0121	0.0206
Digestive diseases	GERD	86	0.0015	0.0270
	IBS*	23	0.0224	0.0234
	cholelithiasis	140	0.0235	0.0332
	AP*	30	0.0027	0.1684
Renal disease	CKD	23	0.0040	0.0091
Eye diseases	cataract	51	0.0045	0.0349
	glaucoma	119	0.0073	0.0674
Respiratory diseases	COPD	18	0.0074	0.0131
	asthma	20	0.0066	0.0112
Cancer	lung cancer	18	0.0065	0.1038
	breast cancer	46	0.0151	0.1231
	prostate cancer	47	0.0052	0.0109
	colon cancer*	42	0.0066	0.2272
	rectal cancer*	41	0.0126	0.3033

Note: Total SNPs: SNPs used to calculate polygenic risk score via the P+T method. *: since no SNPs in these diseases met the criterion ($P<5\times10^{-8}$), the P-value threshold was relaxed to 1×10^{-5}. McFadden R^2 was calculated to assess disease risk. SNP, single nucleotide polymorphism; P+T, pruning and thresholding; PRS-CS, polygenic risk score-continuous shrinkage. PAD, panic/anxiety disorder; BPD, bipolar disorder; MDD, major depressive disorder; SCZ, schizophrenia; AD, Alzheimer's disease; PD, Parkinson disease; CAD, coronary artery disease; AF, Atrial fibrillation; MI, myocardial infarction; HF, heart failure; T2D, type 2 diabetes; IBD, inflammatory bowel disease; CD, Crohn's disease; UC, ulcerative colitis; MS, multiple sclerosis; GERD, gastroesophageal reflux disease; IBS, irritable bowel syndrome; AP, acute pancreatitis; CKD, chronic kidney disease; COPD, chronic obstructive pulmonary disease.

Table S4. Clinical risk tools and included variables for complex diseases.

Category	Diseases	risk score	risk factors	Reference
Neuropsychiatric disorders	PAD	/	age, sex, smoke, alcohol, ACE, cannabis use, social support, physical activity.	[38-40]
	BPD	/	age, sex, history of IBS, ACE, birthweight, cannabis use, psychosocial stress.	[41-43]
	MDD	/	age, sex, BMI, education, family history, social support, diet, sleep, ACE, CRP.	[44-46]
	SCZ	/	age, sex, cannabis use, ACE, social support, psychosocial stress, TDI.	[47-49]
Neurodegenerative diseases	AD	CAIDE	age, sex, education, systolic blood pressure, BMI, total cholesterol, physical activity, APOE4.	[5]
	PD	/	age, sex, smoke, alcohol, physical activity, family history, diet, education, pesticides exposure.	[50-52]
Cardiometabolic diseases	CAD	FRS	age, sex, total and high-density lipoprotein cholesterol, systolic blood pressure, treatment for hypertension, smoking and diabetes status.	[10]
	hypertension	FRS-HPT	age, sex, systolic and diastolic blood pressure, body mass index, parental hypertension, and cigarette smoking.	[6]
	stroke	CHA_2DS_2-VASc	congestive heart failure, hypertension, age, diabetes, prior stroke, vascular disease, and sex.	[7]
	AF	CHARGE-AF	age, height, weight, systolic and diastolic blood pressure, current smoking, use of antihypertensive medication, diabetes, and history of MI and HF.	[8,9]
	MI	FRS	age, sex, total and high-density lipoprotein cholesterol, systolic blood pressure, treatment for hypertension, smoking and diabetes status.	[10]
	HF	FRS	age, sex, total and high-density lipoprotein cholesterol, systolic blood pressure, treatment for hypertension, smoking and diabetes status.	[10]
	T2D	ADA	age, sex, height, weight, history of gestational diabetes, family history of diabetes, hypertension and physical activity.	[11]
Immune diseases	gout	/	age, sex, urate, diet, alcohol, TDI, BMI, physical activity, hypertension, history of CKD.	[53-55]
	IBD	/	age, sex, smoke, alcohol, diet, breastfeeding, antibiotics, physical activity,	[56-58]

	CD	/	Vitamin D.	
	UC	/		
	MS	/	age, sex, BMI, smoke, Vitamin D, diet, shift work.	[59-61]
Digestive diseases	GERD	/	age, sex, BMI, smoke, alcohol, diet, physical activity, TDI.	[62-64]
	IBS	/	age, sex, diet, antibiotics, physical activity, stress.	[65-67]
	cholelithiasis	/	age, sex, BMI, diet, TDI, total cholesterol, physical activity.	[68,69]
	AP	/	age, sex, BMI, smoke, alcohol, history of CHO, total cholesterol.	[70,71]
Renal disease	CKD	/	age, sex, BMI, smoke, alcohol, diabetes, hypertension, physical activity.	[72,73]
Eye diseases	cataract	/	age, sex, smoke, alcohol, TDI, diabetes, hypertension, diet.	[74,75]
	glaucoma	/	age, sex, smoke, alcohol, TDI, history of CAD.	[76-78]
Respiratory diseases	COPD	/	age, sex, smoke, air pollution, diet, TDI, occupational exposures.	[79-81]
	asthma	/	age, sex, birthweight, BMI, occupational exposures, air pollution, diet, TDI.	[81,82]
Cancer	lung cancer	/	age, sex, smoke. air pollution, diet, alcohol, BMI, family history, history of COPD, occupational exposures.	[83-85]
	breast cancer	/	age, BMI, family history, age of menarche, age of menopause, age at first live birth, number of live births, smoke, alcohol.	[86-88]
	prostate cancer	/	age, BMI, smoke, alcohol, family history, diet, TDI, physical activity.	[89-91]
	colon cancer	/	age, sex, BMI, diet, smoke, alcohol, family history, history of IBD, T2D, physical activity, birthweight.	[92-94]
	rectal cancer	/		

Note: PAD, panic/anxiety disorder; BPD, bipolar disorder; MDD, major depressive disorder; SCZ, schizophrenia; AD, Alzheimer's disease; PD, Parkinson disease; CAD, coronary artery disease; AF, Atrial fibrillation; MI, myocardial infarction; HF, heart failure; T2D, type 2 diabetes; IBD, inflammatory bowel disease; CD, Crohn's disease; UC, ulcerative colitis; MS, multiple sclerosis; GERD, gastroesophageal reflux disease; IBS, irritable bowel syndrome; AP, acute pancreatitis; CKD, chronic kidney disease; COPD, chronic obstructive pulmonary disease; ACE, adverse childhood experience; BMI, body mass index; CRP, C-reactive protein; TDI, Townsend deprivation index; APOE4 , apolipoprotein E ε4.

Table S5. Thresholds for early onset age of complex diseases.

Category	Diseases	Early onset age	Reference
Neuropsychiatric disorders	PAD	<20	[95]
	BPD	<18	[96]
	MDD	<20	[97]
	SCZ	<18	[98]
Neurodegenerative diseases	AD	<65	[99]
	PD	<65	[100]
Cardiometabolic diseases	CAD	<55	[101]
	hypertension	<45	[102]
	stroke	<50	[103]
	AF	<60	[104]
	MI	<50	[105]
	HF	<50	[106]
	T2D	<40	[107]
Immune diseases	gout	<40	[108]
	IBD	<18	[109]
	CD	<18	[109]
	UC	<18	[109]
	MS	<18	[110]
Digestive diseases	GERD	<50	[111]
	IBS	<20	[112]
	cholelithiasis	<40	[113]
	AP	<20	[114]
Renal disease	CKD	<50	[115]
Eye diseases	cataract	<50	[116]
	glaucoma	<40	[117]
Respiratory diseases	COPD	<50	[118]
	asthma	<12	[119]
Cancer	lung cancer	<50	[120]
	breast cancer	<40	[121]
	prostate cancer	<55	[122]
	colon cancer	<50	[123]
	rectal cancer	<50	[123]

Note: PAD, panic/anxiety disorder; BPD, bipolar disorder; MDD, major depressive disorder; SCZ, schizophrenia; AD, Alzheimer's disease; PD, Parkinson disease; CAD, coronary artery disease; AF, Atrial fibrillation; MI, myocardial infarction; HF, heart failure; T2D, type 2 diabetes; IBD, inflammatory bowel disease; CD, Crohn's disease; UC, ulcerative colitis; MS, multiple sclerosis; GERD, gastroesophageal reflux disease; IBS, irritable bowel syndrome; AP, acute pancreatitis; CKD, chronic kidney disease; COPD, chronic obstructive pulmonary disease.

Table S6. Baseline characteristics of the included European participants.

**Characteristics**	**Research data set (*N*=455,067)**
Follow-up time, median (IQR)	13.5 (1.3)
Age, mean (SD)	57.3 (8.0)
BMI, mean (SD)	27.4 (4.8)
TDI, mean (SD)	-1.5 (3.0)
SBP, mean (SD)	141.7 (20.7)
Birthweight, mean (SD)	3.3 (0.7)
CRP, mean (SD)	2.6 (4.3)
TC, mean (SD)	5.7 (1.1)
Sleep time, mean (SD)	7.1 (1.2)
ACE, mean (SD)	6.1 (2.6)
Stress score, mean (SD)	-2.7 (5.0)
Urate, mean (SD)	309.8 (80.2)
Vitamin D, mean (SD)	49.2 (21.5)
Air pollution score, mean (SD)	-0.9 (15.8)
Male, *N* (%)	208002 (45.7)
Smoking, *N* (%)	
No	177154 (38.9)
Yes	277913 (61.1)
Drinking, *N* (%)	
No	437860 (96.2)
Yes	17207 (3.8)
Diet score, *N* (%)	
0	8630 (1.9)
1	40667 (8.9)
2	90382 (19.9)
3	125227 (27.5)
4	121841 (26.8)
5	68320 (15.0)
Physical activity, *N* (%)	
low	85894 (18.9)
moderate	186016 (40.9)
high	183157 (40.2)
Education, *N* (%)	
No	308498 (67.8)
Yes	146569 (32.2)
Social support score, *N* (%)	
0	32067 (7.0)
1	143668 (31.6)
2	279332 (61.4)
Cannabis use, *N* (%)	
No	360692 (79.3)
1-2 times	16072 (3.5)
3-10 times	42923 (9.4)
11-100 times	7518 (1.7)
more than 100 times	27862 (6.1)
Pesticide exposure, *N* (%)	
No	432408 (95.0)

Yes	22659 (5.0)
Dust exposure, *N* (%)	
No	224257 (49.3)
Yes	230810 (50.7)
Breastfeeding, *N* (%)	
No	143014 (31.4)
Yes	312053 (68.6)
Antibiotics use, *N* (%)	
No	385693 (84.8)
Yes	69374 (15.2)
Shiftwork, *N* (%)	
No	407357 (89.5)
Yes	47710 (10.5)
History of hypertension, *N* (%)	177293 (39.0)
History of diabetes, *N* (%)	35562 (7.8)
History of IBS, *N* (%)	23961 (5.3)
History of CKD, *N* (%)	5375 (1.2)
History of CAD, *N* (%)	55586 (12.2)
History of COPD, *N* (%)	9022 (2.0)
History of IBD, *N* (%)	5335 (1.2)
History of cholelithiasis, *N* (%)	15179 (3.3)
Family history of MDD, *N* (%)	63047 (13.9)
Family history of PD, *N* (%)	19200 (4.2)
Family history of lung cancer, *N* (%)	60804 (13.4)
Family history of breast cancer, *N* (%)	51257 (11.3)
Family history of prostate cancer, *N* (%)	37870 (8.3)
Family history of colorectal cancer, *N* (%)	53491 (11.8)
ADA, mean (SD)	4.4 (1.7)
CHA_2DS_2-VASc, mean (SD)	1.1 (0.9)
CHARGE-AF, mean (SD)	11.4 (1.0)
FRS-HPT, mean (SD)	37.9 (20.2)
CAIDE, mean (SD)	7.8 (3.6)
FRS, *N* (%)	
0	154358 (33.9)
1	140093 (30.8)
2	160616 (35.3)

Note: IQR, interquartile range; SD, standard deviation; BMI, body mass index; TDI, Townsend deprivation index; SBP, systolic blood pressure; CRP, C-reactive protein; TC, total cholesterol; ACE, adverse childhood experience; MDD, major depressive disorder; PD, Parkinson disease; CAD, coronary artery disease; IBD, inflammatory bowel disease; IBS, irritable bowel syndrome; CKD, chronic kidney disease; COPD, chronic obstructive pulmonary disease; ADA,T2D risk score; CHA_2DS_2-VASc, stroke risk score; CHARGE-AF, AF risk score; FRS-HPT, the Framingham Hypertension Risk Score; CAIDE, the Cardiovascular Risk Factors, Ageing and Dementia risk score; FRS, the Framingham Risk Score.

Table S7. Incident risk association for PRS (per SD) and 32 complex diseases.

Category	Diseases	All incident		Early onset		Late onset	
		**HR (95%CI)**	***P***	**HR (95%CI)**	***P***	**HR (95%CI)**	***P***
Neuropsychiatric disorders	PAD	1.07 (1.06~1.08)	9.61×10^{-25}	/	/	/	/
	BPD	1.46 (1.36~1.56)	4.18×10^{-26}	/	/	/	/
	MDD	1.27 (1.25~1.28)	1.44×10^{-238}	/	/	/	/
	SCZ	1.66 (1.50~1.83)	6.60×10^{-24}	/	/	/	/
Neurodegenerative diseases	AD	1.96 (1.90~2.02)	<0.001	1.60 (1.39~1.84)	4.92×10^{-11}	1.98 (1.92~2.05)	<0.001
	PD	1.33 (1.28~1.38)	1.21×10^{-47}	1.54 (1.37~1.73)	1.24×10^{-12}	1.30 (1.25~1.36)	3.50×10^{-38}
Cardiometabolic diseases	CAD	1.46 (1.44~1.47)	<0.001	1.95 (1.87~2.03)	4.51×10^{-227}	1.43 (1.42~1.45)	<0.001
	hypertension	1.27 (1.26~1.28)	<0.001	2.19 (1.92~2.49)	3.81×10^{-33}	1.27 (1.26~1.28)	<0.001
	stroke	1.49 (1.46~1.52)	<0.001	2.18 (1.84~2.58)	1.15×10^{-19}	1.49 (1.46~1.52)	<0.001
	AF	1.66 (1.64~1.68)	<0.001	2.15 (2.07~2.23)	<0.001	1.63 (1.61~1.65)	<0.001
	MI	1.34 (1.32~1.36)	8.51×10^{-264}	1.91 (1.67~2.18)	6.32×10^{-22}	1.33 (1.31~1.35)	3.13×10^{-250}
	HF	1.49 (1.47~1.51)	<0.001	3.02 (2.53~3.59)	3.63×10^{-35}	1.48 (1.46~1.51)	<0.001
	T2D	1.87 (1.85~1.89)	<0.001	/	/	/	/
Immune diseases	gout	1.37 (1.34~1.39)	4.65×10^{-195}	/	/	/	/
	IBD	1.41 (1.36~1.46)	5.94×10^{-77}	/	/	/	/
	CD	1.48 (1.40~1.57)	1.46×10^{-43}	/	/	/	/
	UC	1.42 (1.36~1.48)	8.00×10^{-61}	/	/	/	/
	MS	1.36 (1.25~1.48)	6.42×10^{-13}	/	/	/	/
Digestive diseases	GERD	1.37 (1.36~1.38)	<0.001	1.73 (1.65~1.82)	9.26×10^{-105}	1.36 (1.35~1.37)	<0.001
	IBS	1.12 (1.10~1.14)	1.10×10^{-27}	/	/	/	/
	cholelithiasis	1.38 (1.36~1.40)	<0.001	/	/	/	/
	AP	4.17 (4.03~4.31)	<0.001	/	/	/	/
Renal disease	CKD	1.23 (1.21~1.24)	1.09×10^{-203}	1.40 (1.23~1.59)	3.55×10^{-7}	1.23 (1.21~1.24)	4.02×10^{-199}
Eye diseases	cataract	1.49 (1.47~1.50)	<0.001	2.61 (2.30~2.97)	9.58×10^{-49}	1.48 (1.47~1.50)	<0.001

	glaucoma	1.78 (1.75~1.81)	<0.001	/		/	
Respiratory diseases	COPD	1.26 (1.24~1.28)	8.28×10^{-186}	1.42 (1.24~1.63)	4.16×10^{-7}	1.26 (1.24~1.28)	4.52×10^{-181}
	asthma	1.13 (1.11~1.16)	1.50×10^{-36}	/		/	
Cancer	lung cancer	1.29 (1.25~1.33)	1.91×10^{-61}	1.63 (1.17~2.28)	4.24×10^{-3}	1.29 (1.25~1.33)	4.24×10^{-60}
	breast cancer	2.16 (2.12~2.21)	<0.001	/		/	
	prostate cancer	2.09 (2.05~2.13)	<0.001	2.84 (2.53~3.20)	5.39×10^{-67}	2.07 (2.04~2.11)	<0.001
	colon cancer	3.32 (3.24~3.41)	<0.001	6.11 (5.16~7.23)	5.38×10^{-98}	3.29 (3.21~3.38)	<0.001
	rectal cancer	3.09 (3.01~3.18)	<0.001	4.73 (4.07~5.50)	1.42×10^{-91}	3.06 (2.98~3.15)	<0.001

Note: *P*<0.001, the *P* value is too small to be precisely displayed; HR, hazard ratio; 95%CI, 95% confidence interval; SD, standard deviation; PAD, panic/anxiety disorder; BPD, bipolar disorder; MDD, major depressive disorder; SCZ, schizophrenia; AD, Alzheimer's disease; PD, Parkinson disease; CAD, coronary artery disease; AF, Atrial fibrillation; MI, myocardial infarction; HF, heart failure; T2D, type 2 diabetes; IBD, inflammatory bowel disease; CD, Crohn's disease; UC, ulcerative colitis; MS, multiple sclerosis; GERD, gastroesophageal reflux disease; IBS, irritable bowel syndrome; AP, acute pancreatitis; CKD, chronic kidney disease; COPD, chronic obstructive pulmonary disease; "/", given the enrollment age of the UK Biobank and the cut-off age for early disease onset, we ultimately examined the impact of PRS on both early-onset and late-onset cases for 16 diseases.

Table S8. Incident risk association for stratified PRS and complex diseases.

Category	Diseases	<2.5		2.5-20		80-97.5		>97.5	
		HR (95%CI)	*P*	HR (95%CI)	*P*	HR (95%CI)	*P*	HR (95%CI)	*P*
Neuropsychiatric disorders	PAD	0.90 (0.82~0.98)	1.63×10^{-2}	0.93 (0.89~0.96)	2.38×10^{-5}	1.07 (1.04~1.11)	5.53×10^{-5}	1.26 (1.17~1.36)	5.53×10^{-5}
	BPD	0.81 (0.48~1.35)	4.12×10^{-1}	0.55 (0.43~0.71)	2.38×10^{-6}	1.48 (1.26~1.75)	3.20×10^{-6}	2.58 (1.93~3.45)	3.20×10^{-6}
	MDD	0.59 (0.52~0.66)	1.23×10^{-17}	0.76 (0.73~0.79)	4.71×10^{-36}	1.30 (1.25~1.34)	2.48×10^{-49}	1.60 (1.49~1.72)	2.48×10^{-49}
	SCZ	0.41 (0.15~1.10)	7.72×10^{-2}	0.41 (0.28~0.60)	7.37×10^{-6}	1.57 (1.26~1.95)	6.09×10^{-5}	2.79 (1.93~4.04)	6.09×10^{-5}
Neurodegenerative diseases	AD	0.45 (0.30~0.67)	1.23×10^{-4}	0.56 (0.48~0.64)	8.90×10^{-15}	2.16 (1.99~2.34)	3.99×10^{-76}	5.19 (4.62~5.82)	3.99×10^{-76}
	PD	0.53 (0.38~0.75)	3.30×10^{-4}	0.77 (0.68~0.87)	1.19×10^{-5}	1.33 (1.21~1.46)	2.67×10^{-9}	1.98 (1.65~2.37)	2.67×10^{-9}
Cardiometabolic diseases	CAD	0.50 (0.45~0.55)	1.04×10^{-44}	0.66 (0.64~0.69)	2.08×10^{-121}	1.62 (1.58~1.66)	<0.001	2.60 (2.48~2.72)	<0.001
	hypertension	0.77 (0.72~0.82)	1.91×10^{-16}	0.81 (0.79~0.83)	1.94×10^{-63}	1.22 (1.20~1.25)	2.79×10^{-89}	1.51 (1.45~1.57)	4.59×10^{-87}
	stroke	0.51 (0.42~0.62)	1.40×10^{-11}	0.72 (0.68~0.77)	1.29×10^{-21}	1.64 (1.56~1.72)	1.17×10^{-90}	3.19 (2.94~3.45)	1.17×10^{-90}
	AF	0.34 (0.30~0.38)	1.23×10^{-4}	0.54 (0.52~0.57)	8.90×10^{-15}	1.93 (1.88~1.98)	3.99×10^{-76}	3.90 (3.74~4.08)	3.99×10^{-76}
	MI	0.51 (0.43~0.59)	3.48×10^{-18}	0.69 (0.66~0.73)	7.36×10^{-42}	1.41 (1.35~1.46)	6.08×10^{-64}	1.89 (1.75~2.04)	6.08×10^{-64}
	HF	0.65 (0.57~0.74)	5.04×10^{-11}	0.71 (0.67~0.74)	2.30×10^{-43}	1.63 (1.57~1.69)	8.06×10^{-156}	3.36 (3.16~3.57)	8.06×10^{-156}
	T2D	0.32 (0.27~0.37)	2.10×10^{-48}	0.48 (0.46~0.51)	7.51×10^{-195}	1.98 (1.93~2.03)	<0.001	3.75 (3.59~3.91)	<0.001
Immune diseases	gout	0.65 (0.52~0.79)	3.09×10^{-5}	0.77 (0.72~0.83)	9.97×10^{-14}	1.20 (1.14~1.26)	1.15×10^{-12}	1.37 (1.24~1.51)	1.15×10^{-12}
	IBD	0.57 (0.41~0.78)	3.87×10^{-4}	0.68 (0.61~0.77)	1.67×10^{-10}	1.62 (1.48~1.76)	1.20×10^{-28}	2.17 (1.84~2.56)	1.20×10^{-28}
	CD	0.51 (0.31~0.85)	9.71×10^{-3}	0.59 (0.49~0.72)	1.21×10^{-7}	1.62 (1.42~1.85)	5.74×10^{-13}	2.61 (2.06~3.31)	5.74×10^{-13}
	UC	0.70 (0.50~0.97)	3.40×10^{-2}	0.73 (0.64~0.83)	3.42×10^{-6}	1.74 (1.58~1.92)	4.32×10^{-29}	2.55 (2.12~3.05)	4.32×10^{-29}
	MS	0.53 (0.25~1.12)	9.41×10^{-2}	0.66 (0.50~0.86)	2.54×10^{-3}	1.35 (1.09~1.65)	4.87×10^{-3}	2.09 (1.42~3.08)	4.87×10^{-3}
Digestive diseases	GERD	0.54 (0.49~0.58)	4.63×10^{-45}	0.69 (0.67~0.71)	1.26×10^{-117}	1.45 (1.42~1.48)	6.49×10^{-213}	1.93 (1.85~2.03)	6.49×10^{-213}
	IBS	0.82 (0.71~0.95)	8.64×10^{-3}	0.87 (0.82~0.92)	2.59×10^{-6}	1.16 (1.10~1.22)	2.82×10^{-8}	1.28 (1.14~1.44)	2.82×10^{-8}
	cholelithiasis	0.57 (0.50~0.65)	2.94×10^{-17}	0.73 (0.69~0.76)	2.94×10^{-40}	1.57 (1.51~1.62)	1.23×10^{-138}	2.26 (2.11~2.42)	1.23×10^{-138}
	AP	0.43 (0.24~0.76)	3.52×10^{-3}	0.51 (0.41~0.63)	2.03×10^{-10}	4.80 (4.36~5.29)	1.88×10^{-219}	36.27 (32.94~39.93)	1.88×10^{-219}
Renal disease	CKD	0.63 (0.57~0.71)	5.48×10^{-17}	0.78 (0.75~0.81)	5.59×10^{-36}	1.27 (1.23~1.31)	4.22×10^{-46}	1.58 (1.48~1.70)	4.22×10^{-46}
Eye diseases	cataract	0.51 (0.47~0.54)	1.97×10^{-76}	0.64 (0.62~0.66)	1.54×10^{-230}	1.77 (1.74~1.81)	<0.001	3.34 (3.22~3.46)	<0.001
	glaucoma	0.37 (0.30~0.45)	1.91×10^{-22}	0.53 (0.50~0.57)	9.44×10^{-75}	2.15 (2.06~2.23)	4.62×10^{-297}	4.50 (4.22~4.80)	4.62×10^{-297}

Respiratory diseases	COPD	0.62 (0.54~0.71)	1.13×10^{-12}	0.77 (0.73~0.81)	6.85×10^{-27}	1.30 (1.25~1.35)	2.35×10^{-40}	1.66 (1.53~1.80)	2.35×10^{-40}
	asthma	0.85 (0.74~0.97)	1.75×10^{-2}	0.86 (0.81~0.91)	1.29×10^{-7}	1.14 (1.09~1.20)	1.57×10^{-7}	1.32 (1.18~1.47)	1.57×10^{-7}
Cancer	lung cancer	0.47 (0.35~0.63)	7.62×10^{-7}	0.75 (0.68~0.83)	8.13×10^{-9}	1.32 (1.23~1.42)	1.41×10^{-13}	1.73 (1.50~2.00)	1.41×10^{-13}
	breast cancer	0.37 (0.29~0.48)	1.25×10^{-14}	0.48 (0.44~0.52)	9.32×10^{-60}	2.75 (2.62~2.88)	<0.001	7.51 (7.02~8.02)	<0.001
	prostate cancer	0.11 (0.07~0.16)	4.36×10^{-28}	0.35 (0.32~0.38)	7.52×10^{-120}	2.50 (2.40~2.61)	<0.001	5.25 (4.91~5.60)	<0.001
	colon cancer	0.71 (0.52~0.96)	2.72×10^{-2}	0.75 (0.67~0.85)	4.06×10-6	1.63 (1.48~1.78)	1.89×10^{-25}	25.65 (23.83~27.60)	1.89×10^{-25}
	rectal cancer	0.57 (0.34~0.96)	3.35×10^{-2}	0.78 (0.65~0.93)	7.20×10-3	1.21 (1.03~1.41)	1.76×10^{-2}	26.73 (23.94~29.84)	1.76×10^{-2}

Note: *P*<0.001, the *P* value is too small to be precisely displayed; HR, hazard ratio; 95%CI, 95% confidence interval; PAD, panic/anxiety disorder; BPD, bipolar disorder; MDD, major depressive disorder; SCZ, schizophrenia; AD, Alzheimer's disease; PD, Parkinson disease; CAD, coronary artery disease; AF, Atrial fibrillation; MI, myocardial infarction; HF, heart failure; T2D, type 2 diabetes; IBD, inflammatory bowel disease; CD, Crohn's disease; UC, ulcerative colitis; MS, multiple sclerosis; GERD, gastroesophageal reflux disease; IBS, irritable bowel syndrome; AP, acute pancreatitis; CKD, chronic kidney disease; COPD, chronic obstructive pulmonary disease.

Table S9. C-index (SE) for predictive performance of PRS on 32 complex diseases.

Category	Diseases	PRS			RISK+COV			PRS+RISK+COV		
		**All**	**Early onset**	**Late onset**	**All**	**Early onset**	**Late onset**	**All**	**Early onset**	**Late onset**
Neuropsychiatric disorders	PAD	0.517 (0.001)	/	/	0.600 (0.001)	/	/	0.601 (0.001)	/	/
	BPD	0.597 (0.008)	/	/	0.615 (0.004)	/	/	0.645 (0.006)	/	/
	MDD	0.564 (0.002)	/	/	0.621 (0.001)	/	/	0.633 (0.001)	/	/
	SCZ	0.641 (0.007)	/	/	0.734 (0.011)	/	/	0.762 (0.008)	/	/
Neurodegenerative diseases	AD	0.700 (0.004)	0.645 (0.010)	0.702 (0.002)	0.735 (0.002)	0.532 (0.013)	0.744 (0.002)	0.779 (0.003)	0.618 (0.008)	0.788 (0.002)
	PD	0.577 (0.004)	0.648 (0.009)	0.566 (0.003)	0.765 (0.003)	0.708 (0.010)	0.804 (0.002)	0.773 (0.003)	0.740 (0.011)	0.810 (0.002)
Cardiometabolic diseases	CAD	0.608 (0.001)	0.684 (0.006)	0.604 (0.001)	0.701 (0.001)	0.652 (0.003)	0.705 (0.001)	0.731 (0.001)	0.721 (0.005)	0.734 (0.001)
	hypertension	0.567 (0.001)	0.694 (0.009)	0.568 (0.001)	0.719 (0.001)	0.695 (0.012)	0.720 (0.001)	0.723 (0.001)	0.754 (0.013)	0.723 (0.001)
	stroke	0.606 (0.001)	0.721 (0.015)	0.608 (0.002)	0.615 (0.001)	0.591 (0.013)	0.620 (0.002)	0.652 (0.001)	0.727 (0.011)	0.657 (0.001)
	AF	0.641 (0.001)	0.705 (0.002)	0.636 (0.001)	0.742 (0.001)	0.665 (0.002)	0.781 (0.001)	0.776 (0.001)	0.755 (0.002)	0.807 (0.001)
	MI	0.581 (0.001)	0.664 (0.010)	0.581 (0.001)	0.722 (0.001)	0.665 (0.009)	0.722 (0.001)	0.739 (0.001)	0.718 (0.009)	0.739 (0.001)
	HF	0.604 (0.002)	0.765 (0.011)	0.604 (0.002)	0.704 (0.001)	0.546 (0.011)	0.704 (0.001)	0.733 (0.001)	0.751 (0.010)	0.733 (0.001)
	T2D	0.672 (0.001)	/	/	0.784 (0.001)	/	/	0.815 (0.001)	/	/
Immune diseases	gout	0.586 (0.001)	/	/	0.863 (0.001)	/	/	0.864 (0.001)	/	/
	IBD	0.597 (0.004)	/	/	0.576 (0.004)	/	/	0.620 (0.004)	/	/
	CD	0.618 (0.004)	/	/	0.553 (0.004)	/	/	0.627 (0.006)	/	/
	UC	0.601 (0.003)	/	/	0.592 (0.003)	/	/	0.632 (0.004)	/	/
	MS	0.591 (0.007)	/	/	0.621 (0.006)	/	/	0.646 (0.003)	/	/
Digestive diseases	GERD	0.590 (0.001)	0.653 (0.003)	0.587 (0.001)	0.593 (0.001)	0.929 (0.001)	0.606 (0.001)	0.624 (0.001)	0.935 (0.001)	0.633 (0.001)
	IBS	0.533 (0.002)	/	/	0.615 (0.002)	/	/	0.620 (0.002)	/	/
	cholelithiasis	0.590 (0.001)	/	/	0.659 (0.001)	/	/	0.680 (0.001)	/	/
	AP	0.835 (0.002)	/	/	0.645 (0.004)	/	/	0.846 (0.002)	/	/
Renal disease	CKD	0.558 (0.001)	0.594 (0.013)	0.558 (0.002)	0.790 (<0.001)	0.944 (0.003)	0.795 (0.001)	0.794 (0.001)	0.946 (0.003)	0.798 (0.001)
Eye diseases	cataract	0.613 (0.001)	0.728 (0.011)	0.612 (0.001)	0.740 (0.001)	0.906 (0.004)	0.742 (0.001)	0.765 (<0.001)	0.930 (0.003)	0.767 (0.001)
	glaucoma	0.658 (0.001)	/	/	0.656 (0.001)	/	/	0.720 (0.001)	/	/
Respiratory diseases	COPD	0.568 (0.001)	0.598 (0.010)	0.568 (0.001)	0.771 (0.001)	0.953 (0.004)	0.775 (0.001)	0.776 (0.001)	0.954 (0.004)	0.779 (0.001)
	asthma	0.536 (0.002)	/	/	0.612 (0.003)	/	/	0.617 (0.002)	/	/

	lung cancer	0.582 (0.002)	0.653 (0.039)	0.578 (0.002)	0.791 (0.002)	0.918 (0.022)	0.794 (0.002)	0.796 (0.001)	0.922 (0.022)	0.798 (0.002)
	breast cancer	0.703 (0.001)	/	/	0.563 (0.002)	/	/	0.711 (0.001)	/	/
Cancer	prostate cancer	0.701 (0.001)	0.787 (0.007)	0.701 (0.001)	0.703 (0.001)	0.855 (0.003)	0.717 (0.002)	0.780 (0.001)	0.908 (0.004)	0.791 (0.002)
	colon cancer	0.723 (0.003)	0.879 (0.020)	0.723 (0.003)	0.682 (0.004)	0.901 (0.015)	0.687 (0.002)	0.771 (0.003)	0.944 (0.012)	0.774 (0.003)
	rectal cancer	0.716 (0.003)	0.932 (0.015)	0.713 (0.006)	0.665 (0.005)	0.898 (0.028)	0.676 (0.003)	0.757 (0.004)	0.960 (0.015)	0.759 (0.006)

Note: C-index, concordance index; SE, standard error; PAD, panic/anxiety disorder; BPD, bipolar disorder; MDD, major depressive disorder; SCZ, schizophrenia; AD, Alzheimer's disease; PD, Parkinson disease; CAD, coronary artery disease; AF, Atrial fibrillation; MI, myocardial infarction; HF, heart failure; T2D, type 2 diabetes; IBD, inflammatory bowel disease; CD, Crohn's disease; UC, ulcerative colitis; MS, multiple sclerosis; GERD, gastroesophageal reflux disease; IBS, irritable bowel syndrome; AP, acute pancreatitis; CKD, chronic kidney disease; COPD, chronic obstructive pulmonary diseases. RISK, clinical risk tools; COV, genotype measurement batch and the first ten genetic principal components; "/", given the enrollment age of the UK Biobank and the cut-off age for early disease onset, we ultimately examined the predictive efficiency of PRS in both early-onset and late-onset cases for 16 diseases.

Table S10. Net reclassification improvement (NRI) of PRS for 32 complex diseases.

Category	Diseases	NRI (95%CI)		
		All	Early onset	Late onset
Neuropsychiatric disorders	PAD	0.009 (0.007~0.011)	/	/
	BPD	0.149 (0.123~0.176)	/	/
	MDD	0.052 (0.048~0.056)	/	/
	SCZ	0.031 (0.005~0.057)	/	/
Neurodegenerative diseases	AD	0.119 (0.104~0.134)	0.235 (0.159~0.311)	0.143 (0.128~0.159)
	PD	0.029 (0.019~0.039)	0.070 (0.042~0.098)	0.033 (0.022~0.043)
Cardiometabolic diseases	CAD	0.127 (0.123~0.131)	0.227 (0.208~0.247)	0.126 (0.123~0.129)
	hypertension	0.013 (0.011~0.015)	0.190 (0.140~0.240)	0.013 (0.011~0.015)
	stroke	0.149 (0.141~0.157)	0.326 (0.249~0.403)	0.025 (0.019~0.031)
	AF	0.091 (0.086~0.095)	0.278 (0.261~0.296)	0.081 (0.076~0.085)
	MI	0.112 (0.107~0.116)	0.180 (0.118~0.243)	0.110 (0.105~0.115)
	HF	0.100 (0.095~0.105)	0.568 (0.467~0.669)	0.098 (0.093~0.103)
	T2D	0.128 (0.123~0.133)	/	/
Immune diseases	gout	0.008 (0.005~0.012)	/	/
	IBD	0.162 (0.148~0.175)	/	/
	CD	0.217 (0.194~0.241)	/	/
	UC	0.141 (0.124~0.158)	/	/
	MS	0.068 (0.043~0.094)	/	/
Digestive diseases	GERD	0.108 (0.104~0.112)	0.012 (0.009~0.014)	0.080 (0.077~0.084)
	IBS	0.026 (0.022~0.030)	/	/
	cholelithiasis	0.091 (0.086~0.096)	/	/
	AP	0.698 (0.676~0.721)	/	/
Renal disease	CKD	0.008 (0.005~0.011)	0.020 (0.000~0.040)	0.009 (0.007~0.012)
Eye diseases	cataract	0.057 (0.054~0.060)	0.069 (0.015~0.122)	0.056 (0.053~0.059)
	glaucoma	0.178 (0.170~0.186)	/	/

Respiratory diseases	COPD	0.016 (0.013~0.020)	0.031 (0.002~0.060)	0.016 (0.013~0.020)
	asthma	0.022 (0.018~0.027)	/	/
Cancer	lung cancer	0.016 (0.009~0.024)	0.120 (0.012~0.228)	0.021 (0.015~0.028)
	breast cancer	0.536 (0.524~0.548)	/	/
	prostate cancer	0.222 (0.213~0.232)	0.119 (0.084~0.154)	0.214 (0.205~0.223)
	colon cancer	0.422 (0.400~0.443)	0.247 (0.086~0.408)	0.410 (0.388~0.432)
	rectal cancer	0.442 (0.407~0.477)	0.420 (0.260~0.580)	0.411 (0.376~0.445)

Note: Net reclassification improvement (NRI) was calculated by comparison of two models: PRS + clinical risk tools vs. clinical risk tools. 95%CI, 95% confidence interval; PAD, panic/anxiety disorder; BPD, bipolar disorder; MDD, major depressive disorder; SCZ, schizophrenia; AD, Alzheimer's disease; PD, Parkinson disease; CAD, coronary artery disease; AF, Atrial fibrillation; MI, myocardial infarction; HF, heart failure; T2D, type 2 diabetes; IBD, inflammatory bowel disease; CD, Crohn's disease; UC, ulcerative colitis; MS, multiple sclerosis; GERD, gastroesophageal reflux disease; IBS, irritable bowel syndrome; AP, acute pancreatitis; CKD, chronic kidney disease; COPD, chronic obstructive pulmonary diseases. RISK, clinical risk tools; COV, genotype measurement batch and the first ten genetic principal components.

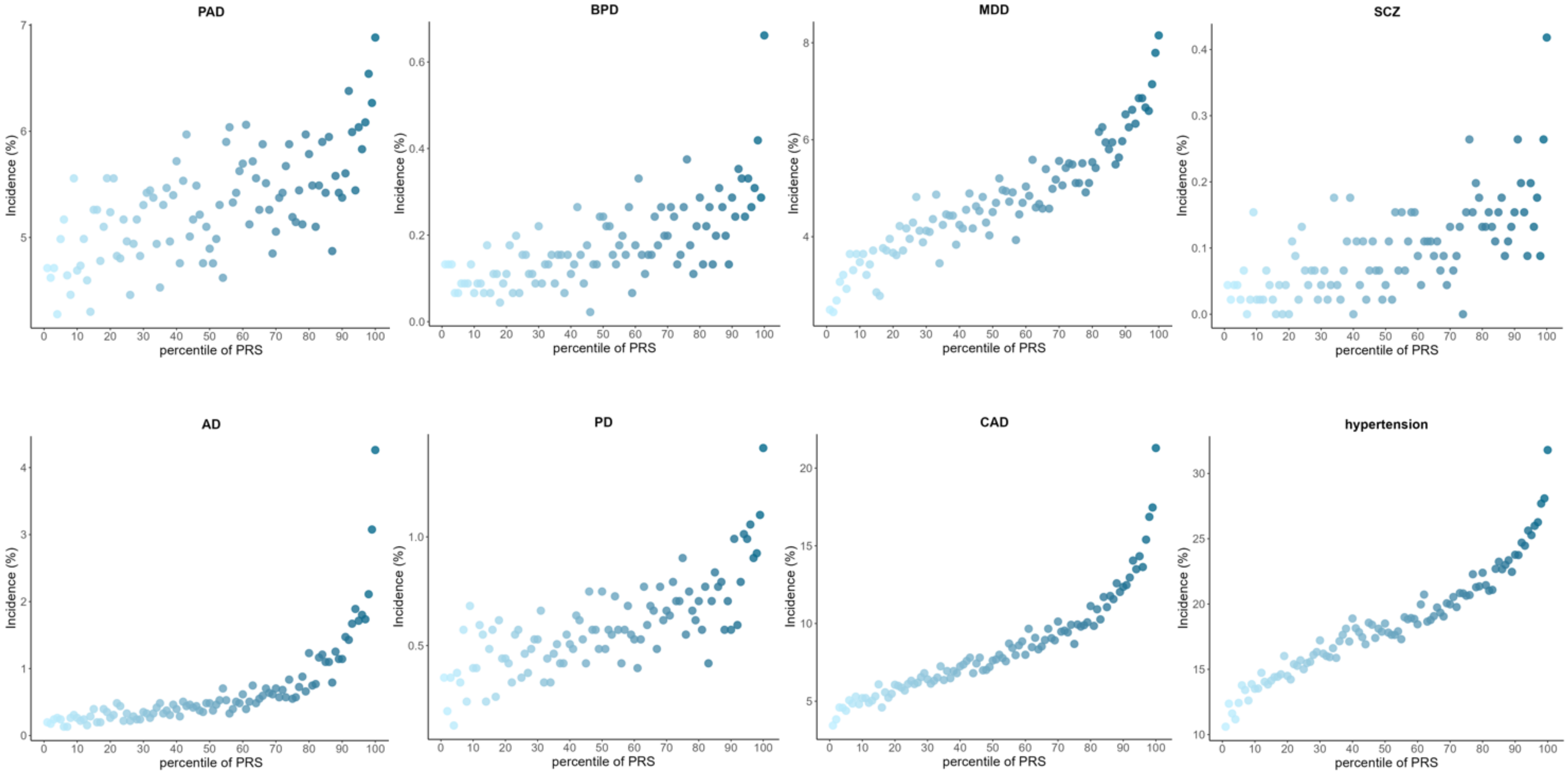
PAD
BPD
MDD
SCZ
AD
PD
CAD
hypertension
Incidence (%)
percentile of PRS
0
10
20
30
40
50
60
70
80
90
100

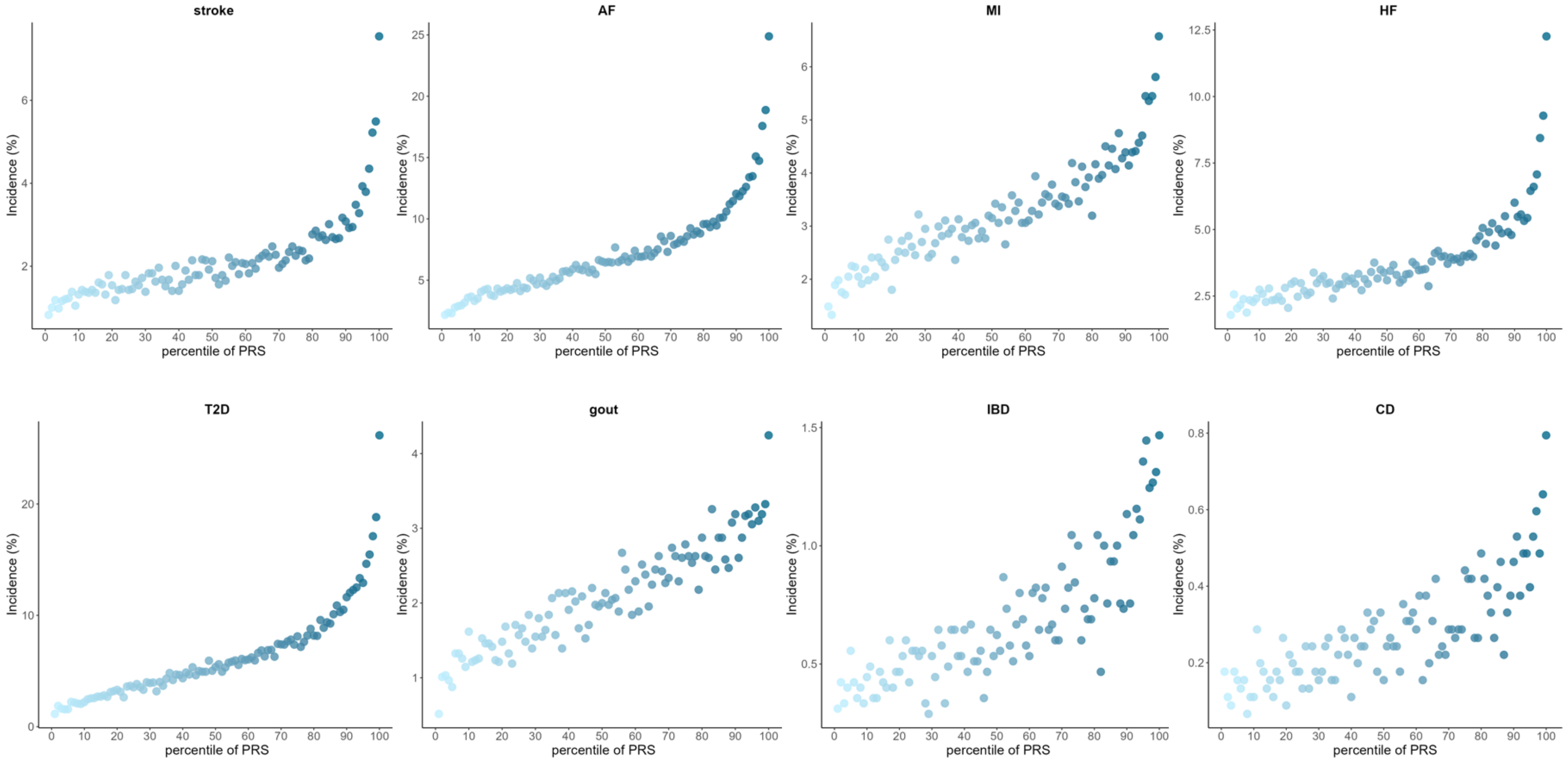
stroke
AF
MI
HF
T2D
gout
IBD
CD
Incidence (%)
percentile of PRS

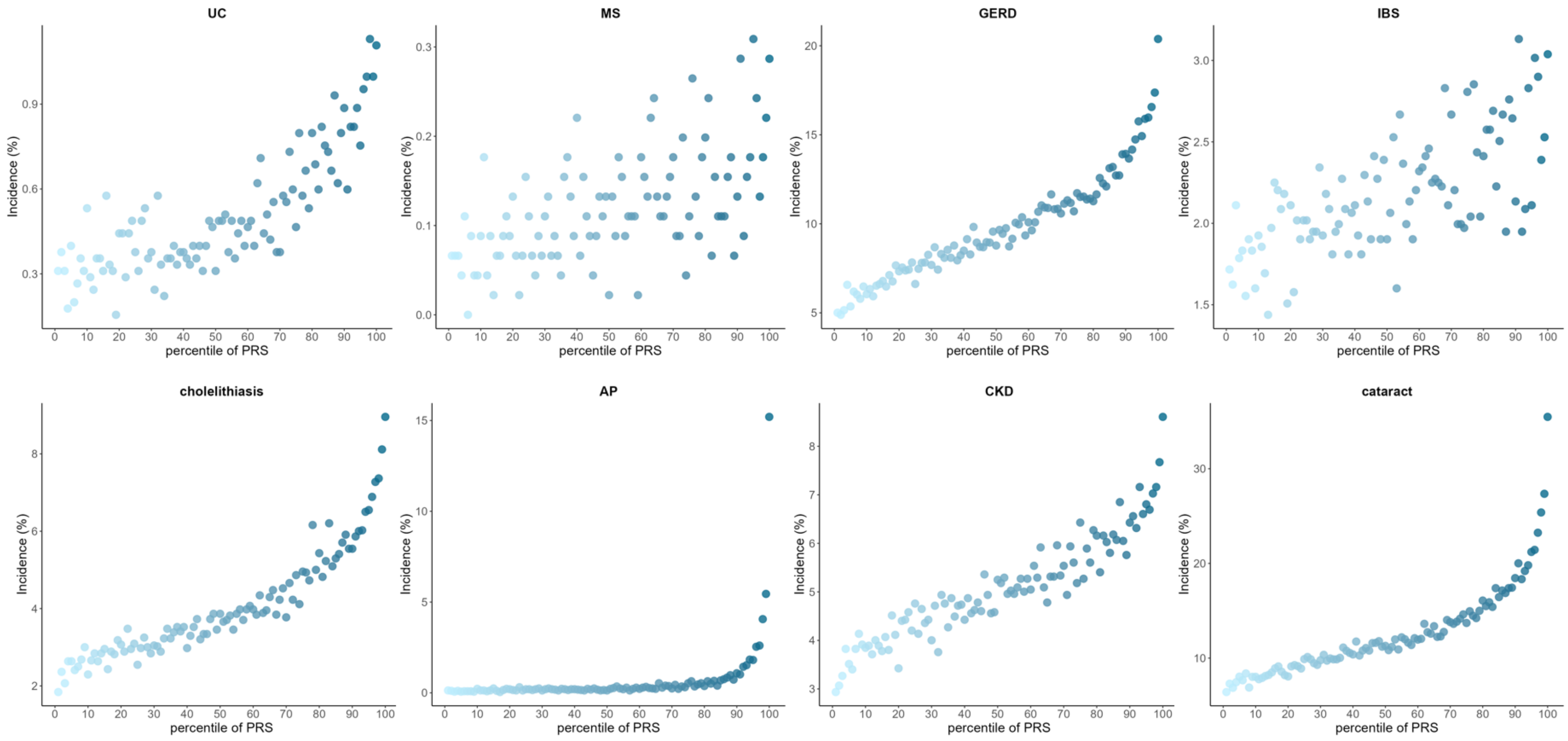
UC
MS
GERD
IBS
cholelithiasis
AP
CKD
cataract
Incidence (%)
percentile of PRS

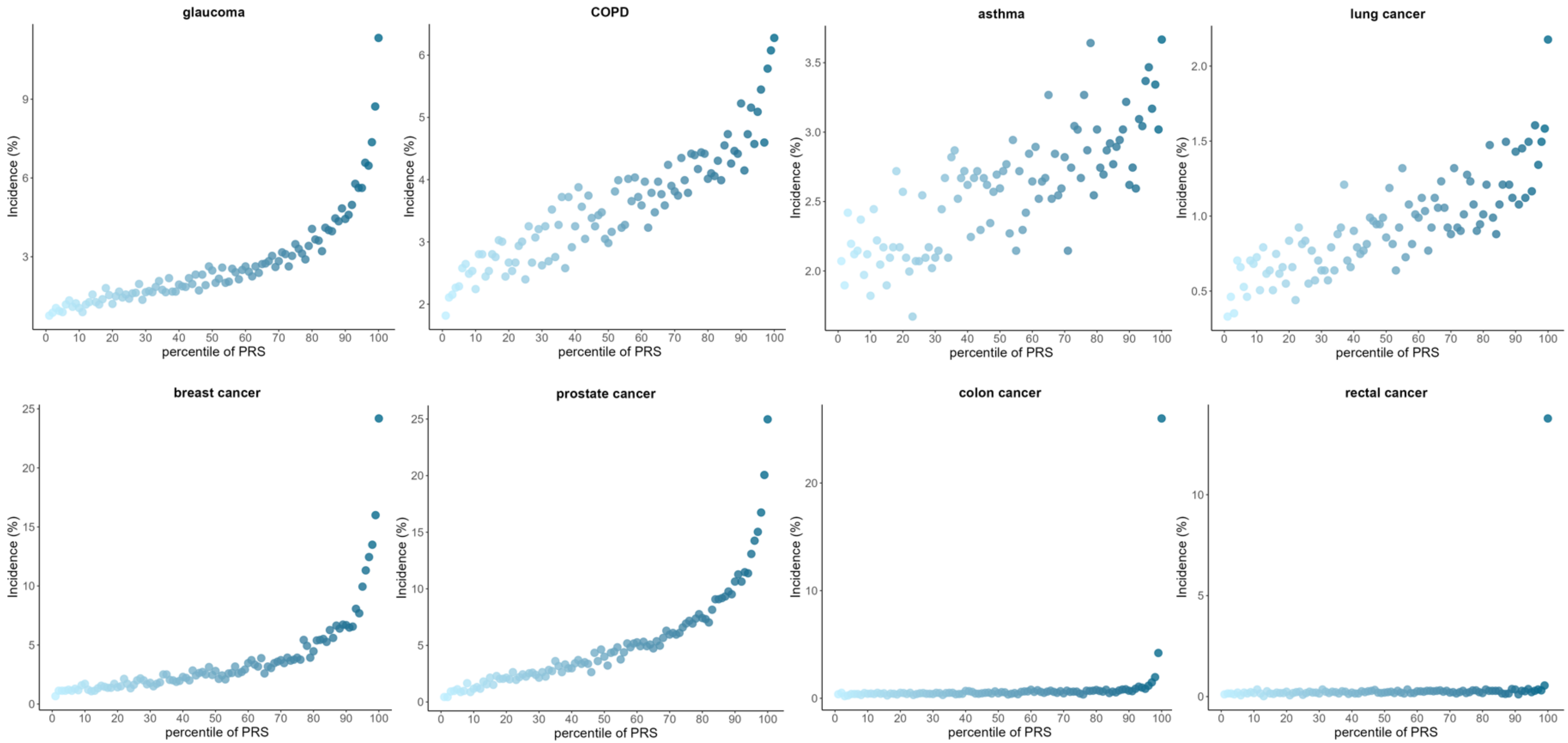

Figure S1. Incidence of complex diseases according to the percentile of the PRS. PAD, panic/anxiety disorder; BPD, bipolar disorder; MDD, major depressive disorder; SCZ, schizophrenia; AD, Alzheimer's disease; PD, Parkinson disease; CAD, coronary artery disease; AF, Atrial fibrillation; MI, myocardial infarction; HF, heart failure; T2D, type 2 diabetes; IBD, inflammatory bowel disease; CD, Crohn's disease; UC, ulcerative colitis; MS, multiple sclerosis; GERD, gastroesophageal reflux disease; IBS, irritable bowel syndrome; AP, acute pancreatitis; CKD, chronic kidney disease; COPD, chronic obstructive pulmonary diseases.

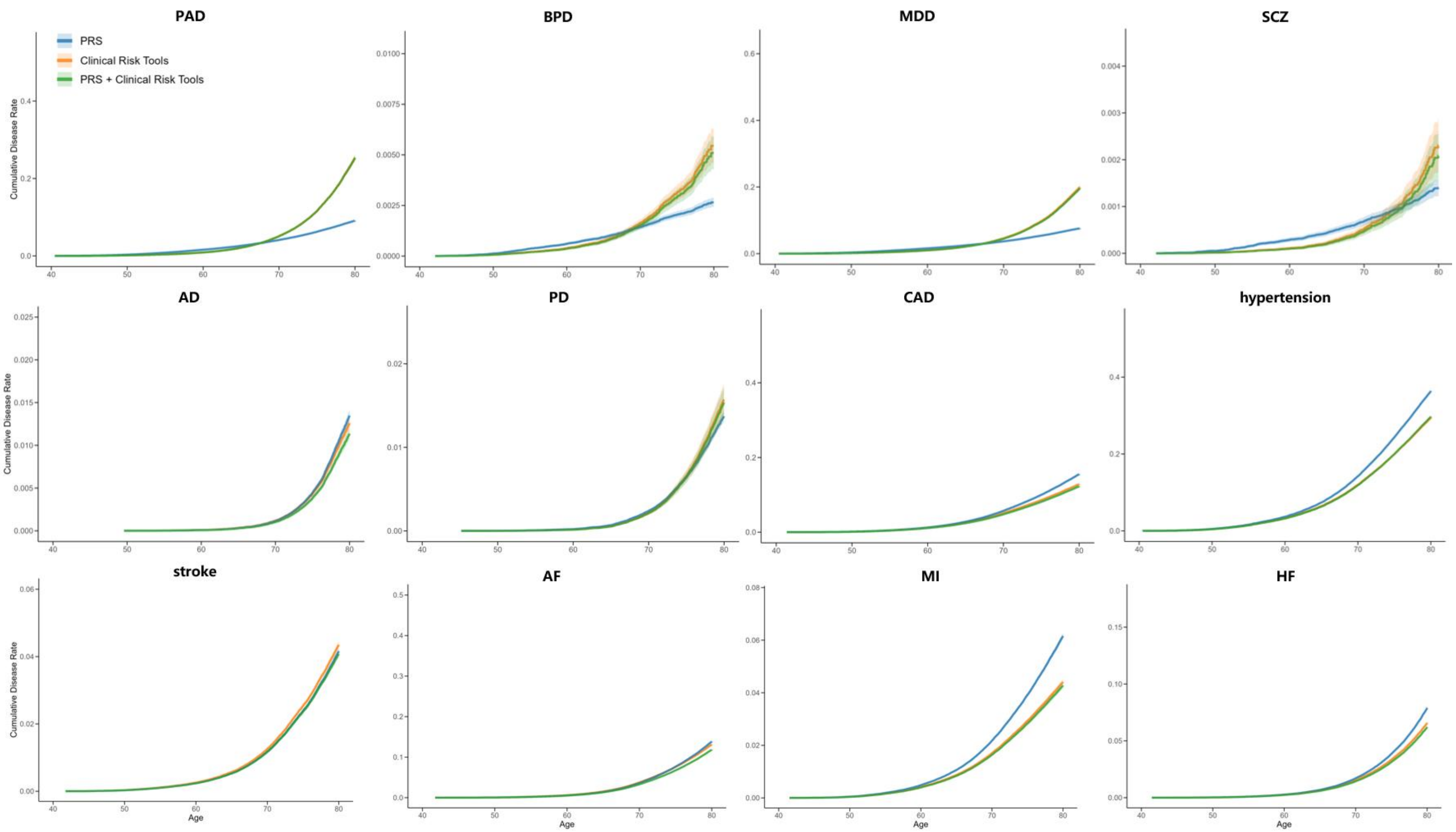
PAD
BPD
MDD
SCZ
AD
PD
CAD
hypertension
stroke
AF
MI
HF
PRS
Clinical Risk Tools
PRS + Clinical Risk Tools
Cumulative Disease Rate
Age

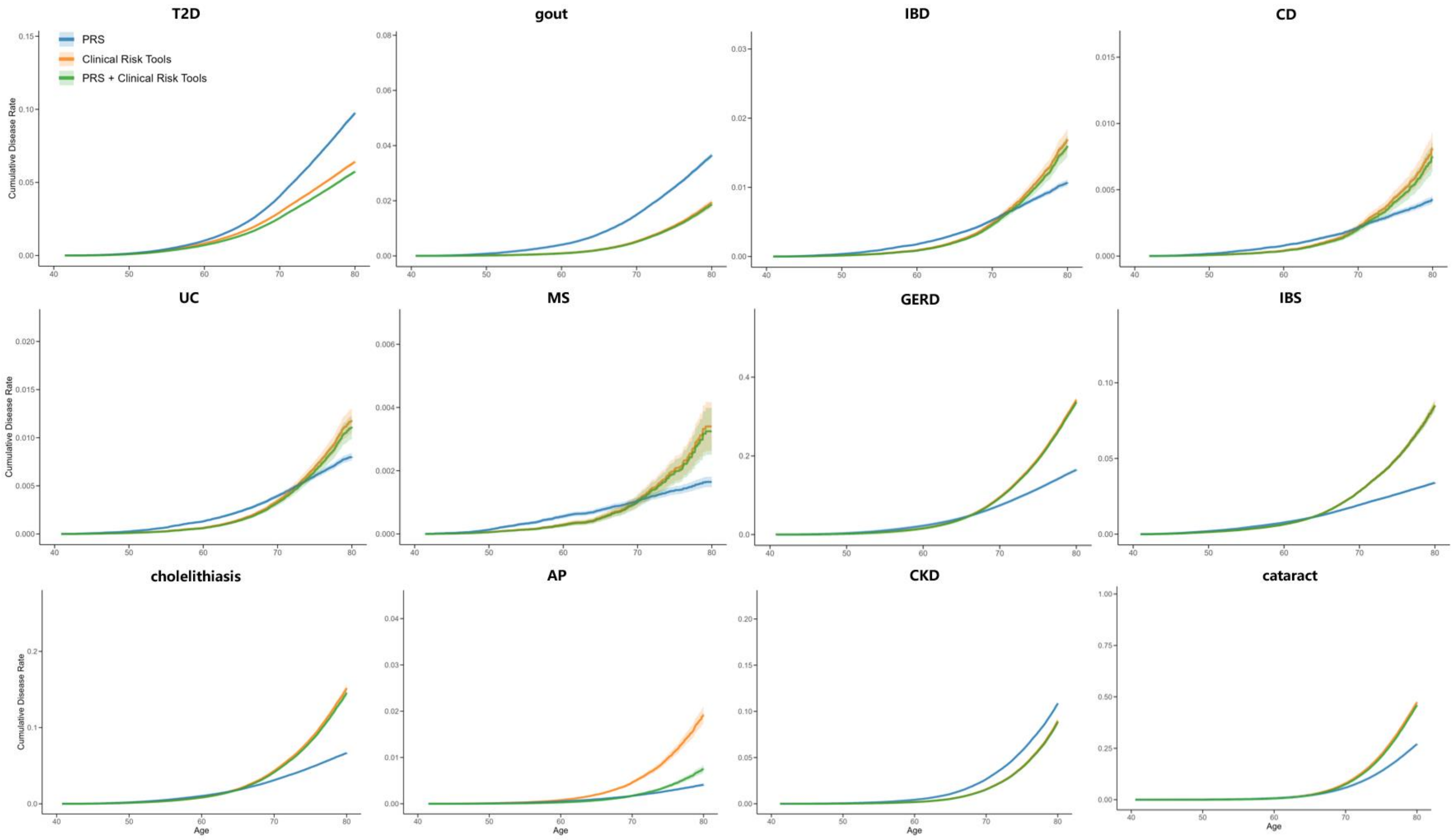
T2D
gout
IBD
CD
UC
MS
GERD
IBS
cholelithiasis
AP
CKD
cataract
PRS
Clinical Risk Tools
PRS + Clinical Risk Tools
Cumulative Disease Rate
Age

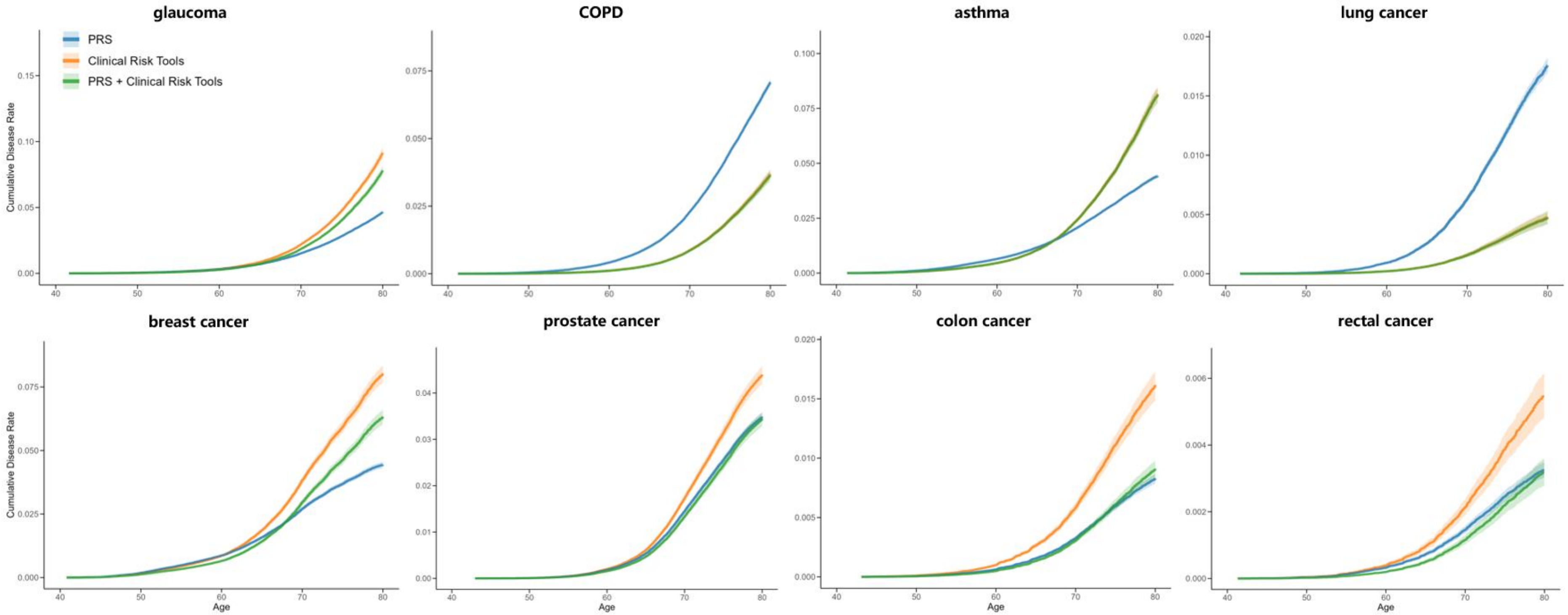

Figure S2. Cumulative incidence of PRS and clinical risk tools. PAD, panic/anxiety disorder; BPD, bipolar disorder; MDD, major depressive disorder; SCZ, schizophrenia; AD, Alzheimer's disease; PD, Parkinson disease; CAD, coronary artery disease; AF, Atrial fibrillation; MI, myocardial infarction; HF, heart failure; T2D, type 2 diabetes; IBD, inflammatory bowel disease; CD, Crohn's disease; UC, ulcerative colitis; MS, multiple sclerosis; GERD, gastroesophageal reflux disease; IBS, irritable bowel syndrome; AP, acute pancreatitis; CKD, chronic kidney disease; COPD, chronic obstructive pulmonary diseases.

References

1 The 1000 Genomes Project Consortium. A global reference for human genetic variation. Nature. 2015;526:68-74.

2 Bulik-Sullivan BK, Loh PR, Finucane HK, Ripke S, Yang J, Patterson N, et al. LD Score regression distinguishes confounding from polygenicity in genome-wide association studies. Nat Genet. 2015;47:291-5.

3 Ge T, Chen CY, Ni Y, Feng YA, Smoller JW. Polygenic prediction via Bayesian regression and continuous shrinkage priors. Nat Commun. 2019;10:1776.

4 Vilhjálmsson BJ, Yang J, Finucane HK, Gusev A, Lindström S, Ripke S, et al. Modeling Linkage Disequilibrium Increases Accuracy of Polygenic Risk Scores. Am J Hum Genet. 2015;97:576-92.

5 Kivipelto M, Ngandu T, Laatikainen T, Winblad B, Soininen H, Tuomilehto J. Risk score for the prediction of dementia risk in 20 years among middle aged people: a longitudinal, population-based study. Lancet Neurol. 2006;5:735-41.

6 Parikh NI, Pencina MJ, Wang TJ, Benjamin EJ, Lanier KJ, Levy D, et al. A risk score for predicting near-term incidence of hypertension: the Framingham Heart Study. Ann Intern Med. 2008;148:102-10.

7 Lip GY, Nieuwlaat R, Pisters R, Lane DA, Crijns HJ. Refining clinical risk stratification for predicting stroke and thromboembolism in atrial fibrillation using a novel risk factor-based approach: the euro heart survey on atrial fibrillation. Chest. 2010;137:263-72.

8 Alonso A, Krijthe BP, Aspelund T, Stepas KA, Pencina MJ, Moser CB, et al. Simple risk model predicts incidence of atrial fibrillation in a racially and geographically diverse population: the CHARGE-AF consortium. J Am Heart Assoc. 2013;2:e000102.

9 Christophersen IE, Yin X, Larson MG, Lubitz SA, Magnani JW, McManus DD, et al. A comparison of the CHARGE-AF and the CHA2DS2-VASc risk scores for prediction of atrial fibrillation in the Framingham Heart Study. Am Heart J. 2016;178:45-54.

10 D'Agostino RB, Sr., Vasan RS, Pencina MJ, Wolf PA, Cobain M, Massaro JM, et al. General cardiovascular risk profile for use in primary care: the Framingham Heart Study. Circulation. 2008;117:743-53.

11 ElSayed NA, Aleppo G, Aroda VR, Bannuru RR, Brown FM, Bruemmer D, et al. 2. Classification and Diagnosis of Diabetes: Standards of Care in Diabetes-2023. Diabetes care. 2023;46:S19-S40.

12 Pencina MJ, D'Agostino RB, Sr., D'Agostino RB, Jr., Vasan RS. Evaluating the added predictive ability of a new marker: from area under the ROC curve to reclassification and beyond. Stat Med. 2008;27:157-72; discussion 207-12.

13 Forstner AJ, Awasthi S, Wolf C, Maron E, Erhardt A, Czamara D, et al. Genome-wide association study of panic disorder reveals genetic overlap with neuroticism and depression. Mol Psychiatry. 2021;26:4179-90.

14 Mullins N, Forstner AJ, O'Connell KS, Coombes B, Coleman JRI, Qiao Z, et al. Genome-wide association study of more than 40,000 bipolar disorder cases

provides new insights into the underlying biology. Nat Genet. 2021;53:817-29.

15 Als TD, Kurki MI, Grove J, Voloudakis G, Therrien K, Tasanko E, et al. Depression pathophysiology, risk prediction of recurrence and comorbid psychiatric disorders using genome-wide analyses. Nat Med. 2023;29:1832-44.

16 Trubetskoy V, Pardiñas AF, Qi T, Panagiotaropoulou G, Awasthi S, Bigdeli TB, et al. Mapping genomic loci implicates genes and synaptic biology in schizophrenia. Nature. 2022;604:502-8.

17 Schwartzentruber J, Cooper S, Liu JZ, Barrio-Hernandez I, Bello E, Kumasaka N, et al. Genome-wide meta-analysis, fine-mapping and integrative prioritization implicate new Alzheimer's disease risk genes. Nat Genet. 2021;53:392-402.

18 Nalls MA, Blauwendraat C, Vallerga CL, Heilbron K, Bandres-Ciga S, Chang D, et al. Identification of novel risk loci, causal insights, and heritable risk for Parkinson's disease: a meta-analysis of genome-wide association studies. Lancet Neurol. 2019;18:1091-102.

19 Aragam KG, Jiang T, Goel A, Kanoni S, Wolford BN, Atri DS, et al. Discovery and systematic characterization of risk variants and genes for coronary artery disease in over a million participants. Nat Genet. 2022;54:1803-15.

20 Zhu Z, Wang X, Li X, Lin Y, Shen S, Liu CL, et al. Genetic overlap of chronic obstructive pulmonary disease and cardiovascular disease-related traits: a large-scale genome-wide cross-trait analysis. Respir Res. 2019;20:64.

21 Mishra A, Malik R, Hachiya T, Jürgenson T, Namba S, Posner DC, et al. Stroke genetics informs drug discovery and risk prediction across ancestries. Nature. 2022;611:115-23.

22 Nielsen JB, Thorolfsdottir RB, Fritsche LG, Zhou W, Skov MW, Graham SE, et al. Biobank-driven genomic discovery yields new insight into atrial fibrillation biology. Nat Genet. 2018;50:1234-9.

23 Nikpay M, Goel A, Won HH, Hall LM, Willenborg C, Kanoni S, et al. A comprehensive 1,000 Genomes-based genome-wide association meta-analysis of coronary artery disease. Nat Genet. 2015;47:1121-30.

24 Shah S, Henry A, Roselli C, Lin H, Sveinbjörnsson G, Fatemifar G, et al. Genome-wide association and Mendelian randomisation analysis provide insights into the pathogenesis of heart failure. Nat Commun. 2020;11:163.

25 Mahajan A, Wessel J, Willems SM, Zhao W, Robertson NR, Chu AY, et al. Refining the accuracy of validated target identification through coding variant fine-mapping in type 2 diabetes. Nat Genet. 2018;50:559-71.

26 Kurki MI, Karjalainen J, Palta P, Sipilä TP, Kristiansson K, Donner KM, et al. FinnGen provides genetic insights from a well-phenotyped isolated population. Nature. 2023;613:508-18.

27 Liu Z, Liu R, Gao H, Jung S, Gao X, Sun R, et al. Genetic architecture of the inflammatory bowel diseases across East Asian and European ancestries. Nat Genet. 2023;55:796-806.

28 Andlauer TF, Buck D, Antony G, Bayas A, Bechmann L, Berthele A, et al. Novel multiple sclerosis susceptibility loci implicated in epigenetic regulation.

Sci Adv. 2016;2:e1501678.

29 Ong JS, An J, Han X, Law MH, Nandakumar P, Schumacher J, et al. Multitrait genetic association analysis identifies 50 new risk loci for gastro-oesophageal reflux, seven new loci for Barrett's oesophagus and provides insights into clinical heterogeneity in reflux diagnosis. Gut. 2022;71:1053-61.

30 Bourgault J, Abner E, Manikpurage HD, Pujol-Gualdo N, Laisk T, Gobeil É, et al. Proteome-Wide Mendelian Randomization Identifies Causal Links Between Blood Proteins and Acute Pancreatitis. Gastroenterology. 2023;164:953-65.e3.

31 Wuttke M, Li Y, Li M, Sieber KB, Feitosa MF, Gorski M, et al. A catalog of genetic loci associated with kidney function from analyses of a million individuals. Nat Genet. 2019;51:957-72.

32 Choquet H, Melles RB, Anand D, Yin J, Cuellar-Partida G, Wang W, et al. A large multiethnic GWAS meta-analysis of cataract identifies new risk loci and sex-specific effects. Nat Commun. 2021;12:3595.

33 Craig JE, Han X, Qassim A, Hassall M, Cooke Bailey JN, Kinzy TG, et al. Multitrait analysis of glaucoma identifies new risk loci and enables polygenic prediction of disease susceptibility and progression. Nat Genet. 2020;52:160-6.

34 Demenais F, Margaritte-Jeannin P, Barnes KC, Cookson WOC, Altmüller J, Ang W, et al. Multiancestry association study identifies new asthma risk loci that colocalize with immune-cell enhancer marks. Nat Genet. 2018;50:42-53.

35 McKay JD, Hung RJ, Han Y, Zong X, Carreras-Torres R, Christiani DC, et al. Large-scale association analysis identifies new lung cancer susceptibility loci and heterogeneity in genetic susceptibility across histological subtypes. Nat Genet. 2017;49:1126-32.

36 Rashkin SR, Graff RE, Kachuri L, Thai KK, Alexeeff SE, Blatchins MA, et al. Pan-cancer study detects genetic risk variants and shared genetic basis in two large cohorts. Nat Commun. 2020;11:4423.

37 Wang A, Shen J, Rodriguez AA, Saunders EJ, Chen F, Janivara R, et al. Characterizing prostate cancer risk through multi-ancestry genome-wide discovery of 187 novel risk variants. Nat Genet. 2023;55:2065-74.

38 Liu Y, Sareen J, Bolton J, Wang J. Development and validation of a risk-prediction algorithm for the recurrence of panic disorder. Depress Anxiety. 2015;32:341-8.

39 Zimmermann M, Chong AK, Vechiu C, Papa A. Modifiable risk and protective factors for anxiety disorders among adults: A systematic review. Psychiatry Res. 2020;285:112705.

40 McNally RJ. Anxiety sensitivity and panic disorder. Biol Psychiatry. 2002;52:938-46.

41 Shintani AO, Rabelo-da-Ponte FD, Marchionatti LE, Watts D, Ferreira de Souza F, Machado CDS, et al. Prenatal and perinatal risk factors for bipolar disorder: A systematic review and meta-analysis. Neurosci Biobehav Rev. 2023;144:104960.

42 Bortolato B, Köhler CA, Evangelou E, León-Caballero J, Solmi M, Stubbs B, et al. Systematic assessment of environmental risk factors for bipolar disorder:

an umbrella review of systematic reviews and meta-analyses. Bipolar Disord. 2017;19:84-96.

43 Marangoni C, Hernandez M, Faedda GL. The role of environmental exposures as risk factors for bipolar disorder: A systematic review of longitudinal studies. J Affect Disord. 2016;193:165-74.

44 Karageorgiou V, Casanova F, O'Loughlin J, Green H, McKinley TJ, Bowden J, et al. Body mass index and inflammation in depression and treatment-resistant depression: a Mendelian randomisation study. BMC Med. 2023;21:355.

45 Lopresti AL, Hood SD, Drummond PD. A review of lifestyle factors that contribute to important pathways associated with major depression: diet, sleep and exercise. J Affect Disord. 2013;148:12-27.

46 Ebert DD, Buntrock C, Mortier P, Auerbach R, Weisel KK, Kessler RC, et al. Prediction of major depressive disorder onset in college students. Depress Anxiety. 2019;36:294-304.

47 Jauhar S, Johnstone M, McKenna PJ. Schizophrenia. Lancet. 2022;399:473-86.

48 Stilo SA, Murray RM. Non-Genetic Factors in Schizophrenia. Curr Psychiatry Rep. 2019;21:100.

49 Chen L, Selvendra A, Stewart A, Castle D. Risk factors in early and late onset schizophrenia. Compr Psychiatry. 2018;80:155-62.

50 Ben-Shlomo Y, Darweesh S, Llibre-Guerra J, Marras C, San Luciano M, Tanner C. The epidemiology of Parkinson's disease. Lancet. 2024;403:283-92.

51 Bloem BR, Okun MS, Klein C. Parkinson's disease. Lancet. 2021;397:2284-303.

52 Periñán MT, Brolin K, Bandres-Ciga S, Blauwendraat C, Klein C, Gan-Or Z, et al. Effect Modification between Genes and Environment and Parkinson's Disease Risk. Ann Neurol. 2022;92:715-24.

53 Dalbeth N, Gosling AL, Gaffo A, Abhishek A. Gout. Lancet. 2021;397:1843-55.

54 Dehlin M, Jacobsson L, Roddy E. Global epidemiology of gout: prevalence, incidence, treatment patterns and risk factors. Nat Rev Rheumatol. 2020;16:380-90.

55 Yip K, Berman J. What Is Gout? JAMA. 2021;326:2541.

56 Saadh MJ, Pal RS, Arias-Gonzáles JL, Orosco Gavilán JC, Jc D, Mohany M, et al. A Mendelian Randomization Analysis Investigates Causal Associations between Inflammatory Bowel Diseases and Variable Risk Factors. Nutrients. 2023;15:1202.

57 Zhang YZ, Li YY. Inflammatory bowel disease: pathogenesis. World J Gastroenterol. 2014;20:91-9.

58 Agrawal M, Allin KH, Petralia F, Colombel JF, Jess T. Multiomics to elucidate inflammatory bowel disease risk factors and pathways. Nat Rev Gastroenterol Hepatol. 2022;19:399-409.

59 Olsson T, Barcellos LF, Alfredsson L. Interactions between genetic, lifestyle and environmental risk factors for multiple sclerosis. Nat Rev Neurol. 2017;13:25-36.

60 Yuan S, Xiong Y, Larsson SC. An atlas on risk factors for multiple sclerosis: a Mendelian randomization study. J Neurol. 2021;268:114-24.

61 Belbasis L, Bellou V, Evangelou E, Ioannidis JP, Tzoulaki I. Environmental risk factors and multiple sclerosis: an umbrella review of systematic reviews and meta-analyses. Lancet Neurol. 2015;14:263-73.

62 Eusebi LH, Ratnakumaran R, Yuan Y, Solaymani-Dodaran M, Bazzoli F, Ford AC. Global prevalence of, and risk factors for, gastro-oesophageal reflux symptoms: a meta-analysis. Gut. 2018;67:430-40.

63 Richter JE, Rubenstein JH. Presentation and Epidemiology of Gastroesophageal Reflux Disease. Gastroenterology. 2018;154:267-76.

64 Katzka DA, Kahrilas PJ. Advances in the diagnosis and management of gastroesophageal reflux disease. BMJ. 2020;371:m3786.

65 Ford AC, Sperber AD, Corsetti M, Camilleri M. Irritable bowel syndrome. Lancet. 2020;396:1675-88.

66 Black CJ, Ford AC. Global burden of irritable bowel syndrome: trends, predictions and risk factors. Nat Rev Gastroenterol Hepatol. 2020;17:473-86.

67 Enck P, Aziz Q, Barbara G, Farmer AD, Fukudo S, Mayer EA, et al. Irritable bowel syndrome. Nat Rev Dis Primers. 2016;2:16014.

68 Chen L, Yang H, Li H, He C, Yang L, Lv G. Insights into modifiable risk factors of cholelithiasis: A Mendelian randomization study. Hepatology. 2022;75:785-96.

69 Lammert F, Gurusamy K, Ko CW, Miquel JF, Méndez-Sánchez N, Portincasa P, et al. Gallstones. Nat Rev Dis Primers. 2016;2:16024.

70 Lankisch PG, Apte M, Banks PA. Acute pancreatitis. Lancet. 2015;386:85-96.

71 Yadav D, Lowenfels AB. The epidemiology of pancreatitis and pancreatic cancer. Gastroenterology. 2013;144:1252-61.

72 Drawz P, Rahman M. Chronic kidney disease. Ann Intern Med. 2015;162:Itc1-16.

73 Hill NR, Fatoba ST, Oke JL, Hirst JA, O'Callaghan CA, Lasserson DS, et al. Global Prevalence of Chronic Kidney Disease - A Systematic Review and Meta-Analysis. PloS one. 2016;11:e0158765.

74 Liu YC, Wilkins M, Kim T, Malyugin B, Mehta JS. Cataracts. Lancet. 2017;390:600-12.

75 Ang MJ, Afshari NA. Cataract and systemic disease: A review. Clin Exp Ophthalmol. 2021;49:118-27.

76 Chan TCW, Bala C, Siu A, Wan F, White A. Risk Factors for Rapid Glaucoma Disease Progression. Am J Ophthalmol. 2017;180:151-7.

77 Stein JD, Khawaja AP, Weizer JS. Glaucoma in Adults-Screening, Diagnosis, and Management: A Review. JAMA. 2021;325:164-74.

78 Quigley HA. Glaucoma. Lancet. 2011;377:1367-77.

79 Yang IA, Jenkins CR, Salvi SS. Chronic obstructive pulmonary disease in never-smokers: risk factors, pathogenesis, and implications for prevention and

treatment. Lancet Respir Med. 2022;10:497-511.

80 Barnes PJ, Burney PG, Silverman EK, Celli BR, Vestbo J, Wedzicha JA, et al. Chronic obstructive pulmonary disease. Nat Rev Dis Primers. 2015;1:15076.

81 Holtjer JCS, Bloemsma LD, Beijers R, Cornelissen MEB, Hilvering B, Houweling L, et al. Identifying risk factors for COPD and adult-onset asthma: an umbrella review. Eur Respir Rev. 2023;32.

82 Miller RL, Grayson MH, Strothman K. Advances in asthma: New understandings of asthma's natural history, risk factors, underlying mechanisms, and clinical management. J Allergy Clin Immunol. 2021;148:1430-41.

83 Malhotra J, Malvezzi M, Negri E, La Vecchia C, Boffetta P. Risk factors for lung cancer worldwide. Eur Respir J. 2016;48:889-902.

84 Leiter A, Veluswamy RR, Wisnivesky JP. The global burden of lung cancer: current status and future trends. Nat Rev Clin Oncol. 2023;20:624-39.

85 Samet JM, Avila-Tang E, Boffetta P, Hannan LM, Olivo-Marston S, Thun MJ, et al. Lung cancer in never smokers: clinical epidemiology and environmental risk factors. Clin Cancer Res. 2009;15:5626-45.

86 Sun YS, Zhao Z, Yang ZN, Xu F, Lu HJ, Zhu ZY, et al. Risk Factors and Preventions of Breast Cancer. Int J Biol Sci. 2017;13:1387-97.

87 Engmann NJ, Golmakani MK, Miglioretti DL, Sprague BL, Kerlikowske K. Population-Attributable Risk Proportion of Clinical Risk Factors for Breast Cancer. JAMA Oncol. 2017;3:1228-36.

88 Kapoor PM, Mavaddat N, Choudhury PP, Wilcox AN, Lindström S, Behrens S, et al. Combined Associations of a Polygenic Risk Score and Classical Risk Factors With Breast Cancer Risk. J Natl Cancer Inst. 2021;113:329-37.

89 Bergengren O, Pekala KR, Matsoukas K, Fainberg J, Mungovan SF, Bratt O, et al. 2022 Update on Prostate Cancer Epidemiology and Risk Factors-A Systematic Review. Eur Urol. 2023;84:191-206.

90 Bostwick DG, Burke HB, Djakiew D, Euling S, Ho SM, Landolph J, et al. Human prostate cancer risk factors. Cancer. 2004;101:2371-490.

91 Gandaglia G, Leni R, Bray F, Fleshner N, Freedland SJ, Kibel A, et al. Epidemiology and Prevention of Prostate Cancer. Eur Urol Oncol. 2021;4:877-92.

92 Patel SG, Karlitz JJ, Yen T, Lieu CH, Boland CR. The rising tide of early-onset colorectal cancer: a comprehensive review of epidemiology, clinical features, biology, risk factors, prevention, and early detection. Lancet Gastroenterol Hepatol. 2022;7:262-74.

93 Gupta S, May FP, Kupfer SS, Murphy CC. Birth Cohort Colorectal Cancer (CRC): Implications for Research and Practice. Clin Gastroenterol Hepatol. 2024;22:455-69.e7.

94 Li N, Lu B, Luo C, Cai J, Lu M, Zhang Y, et al. Incidence, mortality, survival, risk factor and screening of colorectal cancer: A comparison among China, Europe, and northern America. Cancer Lett. 2021;522:255-68.

95 Ramsawh HJ, Weisberg RB, Dyck I, Stout R, Keller MB. Age of onset, clinical

characteristics, and 15-year course of anxiety disorders in a prospective, longitudinal, observational study. J Affect Disord. 2011;132:260-4.

96 Propper L, Ortiz A, Slaney C, Garnham J, Ruzickova M, Calkin CV, et al. Early-onset and very-early-onset bipolar disorder: distinct or similar clinical conditions? Bipolar Disord. 2015;17:814-20.

97 Blank TS, Meyer BM, Wieser MK, Rabl U, Schögl P, Pezawas L. Brain morphometry and connectivity differs between adolescent- and adult-onset major depressive disorder. Depress Anxiety. 2022;39:387-96.

98 Dor-Nedonsel E, Fernandez A, Menard ML, Manera V, Laure G, Thümmler S, et al. Early-onset schizophrenia: studying the links between cognitive and clinical dimensions. Cogn Neuropsychiatry. 2023;28:377-90.

99 van der Flier WM, Pijnenburg YA, Fox NC, Scheltens P. Early-onset versus late-onset Alzheimer's disease: the case of the missing APOE ε4 allele. Lancet Neurol. 2011;10:280-8.

100 Riboldi GM, Frattini E, Monfrini E, Frucht SJ, Di Fonzo A. A Practical Approach to Early-Onset Parkinsonism. J Parkinsons Dis. 2022;12:1-26.

101 Coto E, Reguero JR, Avanzas P, Pascual I, Martín M, Hevia S, et al. Gene variants in the NF-KB pathway (NFKB1, NFKBIA, NFKBIZ) and risk for early-onset coronary artery disease. Immunol Lett. 2019;208:39-43.

102 Niiranen TJ, McCabe EL, Larson MG, Henglin M, Lakdawala NK, Vasan RS, et al. Heritability and risks associated with early onset hypertension: multigenerational, prospective analysis in the Framingham Heart Study. BMJ. 2017;357:j1949.

103 Jiang B, Ryan KA, Hamedani A, Cheng Y, Sparks MJ, Koontz D, et al. Prothrombin G20210A mutation is associated with young-onset stroke: the genetics of early-onset stroke study and meta-analysis. Stroke. 2014;45:961-7.

104 Yoneda ZT, Anderson KC, Quintana JA, O'Neill MJ, Sims RA, Glazer AM, et al. Early-Onset Atrial Fibrillation and the Prevalence of Rare Variants in Cardiomyopathy and Arrhythmia Genes. JAMA Cardiol. 2021;6:1371-9.

105 Do R, Stitziel NO, Won HH, Jørgensen AB, Duga S, Angelica Merlini P, et al. Exome sequencing identifies rare LDLR and APOA5 alleles conferring risk for myocardial infarction. Nature. 2015;518:102-6.

106 Sulo G, Igland J, Nygård O, Vollset SE, Ebbing M, Cerqueira C, et al. Trends in the risk of early and late-onset heart failure as an adverse outcome of acute myocardial infarction: A Cardiovascular Disease in Norway project. Eur J Prev Cardiol. 2017;24:971-80.

107 Liu JJ, Liu S, Wang J, Lee J, Tang JI, Gurung RL, et al. Risk of Incident Heart Failure in Individuals With Early-Onset Type 2 Diabetes. J Clin Endocrinol Metab. 2022;107:e178-e87.

108 Zaidi F, Narang RK, Phipps-Green A, Gamble GG, Tausche AK, So A, et al. Systematic genetic analysis of early-onset gout: ABCG2 is the only associated locus. Rheumatology (Oxford). 2020;59:2544-9.

109 Nambu R, Warner N, Mulder DJ, Kotlarz D, McGovern DPB, Cho J, et al. A Systematic Review of Monogenic Inflammatory Bowel Disease. Clin

Gastroenterol Hepatol. 2022;20:e653-e63.

110 Spiro DB. Early onset multiple sclerosis: a review for nurse practitioners. J Pediatr Health Care. 2012;26:399-408.

111 Wang SE, Kendall BJ, Hodge AM, Dixon-Suen SC, Dashti SG, Makalic E, et al. Demographic and lifestyle risk factors for gastroesophageal reflux disease and Barrett's esophagus in Australia. Dis Esophagus. 2022;35:doab058.

112 Simon E, Călinoiu LF, Mitrea L, Vodnar DC. Probiotics, Prebiotics, and Synbiotics: Implications and Beneficial Effects against Irritable Bowel Syndrome. Nutrients. 2021;13:2112.

113 Denk GU, Bikker H, Lekanne Dit Deprez RH, Terpstra V, van der Loos C, Beuers U, et al. ABCB4 deficiency: A family saga of early onset cholelithiasis, sclerosing cholangitis and cirrhosis and a novel mutation in the ABCB4 gene. Hepatol Res. 2010;40:937-41.

114 Giefer MJ, Lowe ME, Werlin SL, Zimmerman B, Wilschanski M, Troendle D, et al. Early-Onset Acute Recurrent and Chronic Pancreatitis Is Associated with PRSS1 or CTRC Gene Mutations. J Pediatr. 2017;186:95-100.

115 Tsur AM, Akavian I, Landau R, Derazne E, Tzur D, Vivante A, et al. Adolescent Body Mass Index and Early Chronic Kidney Disease in Young Adulthood. JAMA Pediatr. 2024;178:142-50.

116 Papadopoulos C, Kekou K, Xirou S, Kitsiou-Tzeli S, Kararizou E, Papadimas GK. Early onset posterior subscapular cataract in a series of myotonic dystrophy type 2 patients. Eye (Lond). 2018;32:622-5.

117 Selvan H, Gupta S, Wiggs JL, Gupta V. Juvenile-onset open-angle glaucoma - A clinical and genetic update. Surv Ophthalmol. 2022;67:1099-117.

118 Soriano JB, Polverino F, Cosio BG. What is early COPD and why is it important? Eur Respir J. 2018;52:1801448.

119 Yeh JJ, Wang YC, Hsu WH, Kao CH. Incident asthma and Mycoplasma pneumoniae: A nationwide cohort study. J Allergy Clin Immunol. 2016;137:1017-23.e6.

120 Etzel CJ, Lu M, Merriman K, Liu M, Vaporciyan A, Spitz MR. An epidemiologic study of early onset lung cancer. Lung Cancer. 2006;52:129-34.

121 Rantala JNJ, Heikkinen SMM, Hirvonen EM, Tanskanen T, Malila NK, Pitkäniemi JM. Familial aggregation of early-onset cancers in early-onset breast cancer families. Int J Cancer. 2023;153:331-40.

122 Gerhauser C, Favero F, Risch T, Simon R, Feuerbach L, Assenov Y, et al. Molecular Evolution of Early-Onset Prostate Cancer Identifies Molecular Risk Markers and Clinical Trajectories. Cancer cell. 2018;34:996-1011.e8.

123 Eng C, Jácome AA, Agarwal R, Hayat MH, Byndloss MX, Holowatyj AN, et al. A comprehensive framework for early-onset colorectal cancer research. Lancet Oncol. 2022;23:e116-e28.
